# *In vitro* Interactions of *Pseudomonas aeruginosa* With *Scedosporium* Species Frequently Associated With Cystic Fibrosis

**DOI:** 10.3389/fmicb.2019.00441

**Published:** 2019-03-06

**Authors:** Mónika Homa, Alexandra Sándor, Eszter Tóth, Csilla Szebenyi, Gábor Nagy, Csaba Vágvölgyi, Tamás Papp

**Affiliations:** ^1^MTA-SZTE “Lendület" Fungal Pathogenicity Mechanisms Research Group, Szeged, Hungary; ^2^Department of Microbiology, Faculty of Science and Informatics, University of Szeged, Szeged, Hungary; ^3^Interdisciplinary Excellence Centre, Department of Microbiology, University of Szeged, Szeged, Hungary

**Keywords:** *Scedosporium*, *Pseudomonas aeruginosa*, cystic fibrosis, volatile organic compounds, diffusible signal factor, pyocyanin, tobramycin, flucloxacillin

## Abstract

Members of the *Scedosporium apiospermum* species complex are the second most frequently isolated pathogens after *Aspergillus fumigatus* from cystic fibrosis (CF) patients with fungal pulmonary infections. Even so, the main risk factors for the infection are unrevealed. According to previous studies, bacterial infections might reduce the risk of a fungal infection, but an antibacterial therapy may contribute to the airway colonization by several fungal pathogens. Furthermore, corticosteroids, which are often used to reduce lung inflammation in children and adults with CF, are also proved to enhance the growth of *A. fumigatus in vitro*. Considering all the above discussed points, we aimed to test how *Pseudomonas aeruginosa* influences the growth of scedosporia and to investigate the potential effect of commonly applied antibacterial agents and corticosteroids on *Scedosporium* species. Direct interactions between fungal and bacterial strains were tested using the disk inhibition method. Indirect interactions via volatile compounds were investigated by the plate-in-plate method, while the effect of bacterial media-soluble molecules was tested using a modified cellophane assay and also in liquid culture media conditioned by *P. aeruginosa*. To test the effect of bacterial signal molecules, antibacterial agents and corticosteroids on the fungal growth, the broth microdilution method was used. We also investigated the germination ability of *Scedosporium* conidia in the presence of pyocyanin and diffusible signal factor by microscopy. According to our results, *P. aeruginosa* either inhibited or enhanced the growth of scedosporia depending on the culture conditions and the mode of interactions. When the two pathogens were cultured physically separately from each other in the plate-in-plate tests, the presence of the bacteria was able to stimulate the growth of several fungal isolates. While in direct physical contact, bacterial strains inhibited the fungal growth. This effect might be attributed to bacterial signal molecules, which also proved to inhibit the germination and growth of scedosporia. In addition, antibacterial agents showed growth-promoting, while corticosteroids exhibited growth inhibitory effect on several *Scedosporium* isolates. These data raise the possibility that a *P. aeruginosa* infection or a previously administered antibacterial therapy might be able to increase the chance of a *Scedosporium* colonization in a CF lung.

## Introduction

*Scedosporium* species are ubiquitous saprophytic hyalohyphomycetes present in environments affected and transformed by anthropogenic activities, such as agricultural and industrial soils or sewage. Members of the genus *Scedosporium* are associated with a wide spectrum of human infections, including pneumonia, mycetoma, and keratitis in immunocompetent hosts and systemic invasive diseases in immunocompromised patients or in near-drowning victims (Guarro et al., 2006; Cortez et al., 2008). They may also cause usually fatal central nervous system infections in both healthy and immunocompromised individuals through hematogenous spreading from a localized infection or after a surgery (Kowacs et al., 2004; Tortorano et al., 2014) and are frequently detected in the sputum of patients with cystic fibrosis (CF) (Schwarz et al., 2017).

Molecular identification methods and the introduction of the new nomenclature system for fungi (Hawksworth et al., 2011) resulted in fundamental changes in the taxonomy of the related genera *Scedosporium* and *Pseudallescheria*. For instance, the phylogenetically and morphologically distinct but clinically relevant *Scedosporium prolificans* has been reallocated to the genus *Lomentospora* (Lackner et al., 2014). Currently, the genus *Scedosporium* is considered to comprise 10 species, namely *S. aurantiacum*, *S. cereisporum*, *S. dehoogii*, *S. desertorum*, *S. minutisporum* and the members of the *S. apiospermum* species complex, including *S. angustum*, *S. apiospermum*, *S. boydii*, *S. ellipsoideum* and *S. fusoideum* (Ramirez-Garcia et al., 2018). Out of these species, *S. apiospermum*, *S. boydii*, and *S. aurantiacum* are regularly, while *S. ellipsoideum* and *S. minutisporum* are sporadically associated with CF lung infections with a geographically variable prevalence (Blyth et al., 2010; Zouhair et al., 2013; Sedlacek et al., 2015). *S. angustum* is not a common CF pathogen, but it has been reported from human lung mycetoma before (Kravitz et al., 2011).

Cystic fibrosis is one of the most common genetic disorders, that is resulted by the mutation of the gene encoding the CF transmembrane conductance regulator (CFTR) protein. Dysfunction of CFTR affects the transepithelial fluid and the ion transport of the exocrine glands and leads to a nutrient rich, thick airway surface liquid in the lungs. This highly viscous mucus cannot be cleared effectively by the muco-ciliary transport system supporting the colonization of the airways by bacterial and fungal pathogens (Lightly et al., 2017). Chronic infections are among the primary causes of mortality of CF patients, since both the infecting microbes and the inflammation response cause irreversible damage in the lung tissue and lead to a respiratory failure (LiPuma, 2010).

Generally, *Staphylococcus aureus* is the first colonizer of the CF lung, but by the time the patients reach the adulthood, *P. aeruginosa* becomes the most frequently isolated bacterium causing chronic lung infections (LiPuma, 2010). Among filamentous fungi, *Aspergillus fumigatus* (16–58%) and the *S. apiospermum* species complex (9–10%) are the most prevalent pathogens isolated from CF patients (Noni et al., 2017; Reece et al., 2017). Clinical manifestations and radiological and histopathological appearance of *Scedosporium* may overlap with other hyaline molds (i.e., *Aspergillus* and *Fusarium* spp.) (Rolfe et al., 2013; Ramirez-Garcia et al., 2018). Moreover, the use of a selective medium would be advantageous for the reliable culture detection of *Scedosporium* spp. in clinical practice since the members of this genus are easily overgrown by rapidly and extensively growing fungi (e.g., *Aspergillus* spp.) giving false negative results on standard culture media (Pham et al., 2015; Sedlacek et al., 2015). Therefore, the real incidence of *Scedosporium* species in CF might be underestimated. The antifungal susceptibility profile of scedosporia is reported to substantially differ from those of other filamentous fungi, such as aspergilli, being less sensitive to the most commonly used antifungal drugs (Guarro et al., 2006; Wiederhold and Lewis, 2009). Consequently, the misdiagnosis of scedosporia might lead to an ineffective therapy and unexpected complications.

While CF-related therapeutic approaches and research have mainly focused on one pathogen at a time, it is more commonly accepted that these infections are associated with dynamically changing polymicrobial communities (Sibley and Surette, 2011). In these communities, both pathogens and commensals might be found. Although the commensal species are mainly harmless, they might affect the infection through their interactions with other members of the community (Noni et al., 2017). The role of scedosporia in the CF-lung microbiota is not yet completely revealed, but its understanding might help in the fight against infections and to improve the patients’ medical conditions.

Risk factors for *Scedosporium* infections in CF patients are still unrevealed. According to Blyth et al. (2010), Australian patients colonized with mucoid *P. aeruginosa* are less frequently infected with scedosporia. A recent study from Germany showed quite the contrary reporting a positive correlation between the co-colonization with mucoid *P. aeruginosa* and *S. apiospermum* complex*/L. prolificans* (Schwarz et al., 2017). Anyway, the co-incidence of *Scedosporium* species with mucoid and non-mucoid *P. aeruginosa* is relatively high, reported to be 75–88.5% and 50–57.7%, respectively (Blyth et al., 2010; Schwarz et al., 2017). Blyth et al. (2010) also reported that antistaphylococcal penicillin therapy may contribute to the airway colonization by scedosporia. Similarly, Burns et al. (1999) found significant correlation between tobramycin therapy and the incidence of *Aspergillus* and *Candida* infections. Interestingly, the volatilome of *P. aeruginosa* was reported to act as a growth-enhancer for aspergilli (Briard et al., 2016), suggesting that a prior bacterial infection might also favor the invasion of the lung by filamentous fungi.

In view of the literature described above, our main purpose was to investigate how *P. aeruginosa* influences fungal growth by indirect and direct physical contact with scedosporia. Besides, we also aimed to test the direct effect of certain antibacterial agents and corticosteroids on the growth of *Scedosporium* species.

## Materials and Methods

### Strains and Culture Conditions

Experiments were performed on two *Pseudomonas aeruginosa* and ten *Scedosporium* isolates representing the species *S. angustum*, *S. apiospermum*, *S. aurantiacum*, *S. boydii*, and *S. ellipsoideum* (Table 1). The two *P. aeruginosa* strains were selected based on their phenotypic differences on lysogeny broth (LB) and their distinct source of isolation. While the strain ATCC 27853 originated from a blood specimen and displayed a yellow colony color, the other strain (ATCC 19429) was derived from a urine sample and formed a dark greenish-blue colony secreting bluish pigments (probably the mixture of pyoverdine, pyorubin, and pyocyanin; Yabuuchi and Ohyama, 1972) in the medium. Since *Aspergillus fumigatus* is a relatively well described CF-related pathogen, we included its clinical isolate SZMC 23245 in all experiments as a reference strain.

**Table 1:** Fungal and bacterial strains involved in the study.

Strain no.	Species	Source
CBS 254.72	*Scedosporium angustum*	Sewage/United States
CBS 117410	*Scedosporium boydii*	Soil/Spain
CBS 117432	*Scedosporium boydii*	CF sputum/France
CBS 120157	*Scedosporium boydii*	Lung, leukemia/France
CBS 301.79	*Scedosporium ellipsoideum*	Dung of cow/Netherlands
CBS 116910	*Scedosporium aurantiacum*	Wound exudate, ulcer of ankle/Spain
CBS 136046	*Scedosporium aurantiacum*	Invasive human lung infection/Australia
CBS 136047	*Scedosporium aurantiacum*	Soil/Australia
CBS 136049	*Scedosporium aurantiacum*	Soil/Austria
SZMC 23374	*Scedosporium apiospermum*	Mycetoma/Hungary
SZMC 23245	*Aspergillus fumigatus*	Keratitis/Croatia
ATCC 27853	*Pseudomonas aeruginosa*	Blood culture/United States
ATCC 19429	*Pseudomonas aeruginosa*	Urine

LB medium (Table 2) was used to culture the bacteria at 37°C for 24 h, while fungal strains were grown on malt-extract agar (MEA, Table 2) at 37°C for 7 days. Both bacterial cells and fungal conidia were harvested freshly before each experiment in saline solution (0.85% NaCl solution supplemented with 0.01% Tween 80). Cell and conidia densities were determined using a Bürker-chamber and the final inoculum densities were set to 10^5^ CFU/ml for the bacterial and 10^4^ CFU/ml for the fungal isolates.

**Table 2:** The list and composition of the media used in the study.

Media	Components
LB	1% NaCl, 1% peptone, 0.5% yeast extract (supplemented with 2% agar in case of solid media)
MEA	5% malt extract, 1% glucose, 2% agar
SCFM (Kozlowska et al., 2013)	*Basic components* (*autoclaved*): 0.65% 0.2 M NaH_2_PO_4_; 0.625% 0.2 M Na_2_HPO_4_; 0.035% 1 M KNO_3_; 1% 119 mM diethylene triamine pentaacetic (Sigma-Aldrich); 1% porcine gastric mucin (Sigma-Aldrich), 0.14% herring sperm DNA (Sigma-Aldrich), 0.2% MOPS (PanReac AppliChem), 0.3% NaCl; 0.012% NH_4_Cl; 0.1% KCl; 2% agar, pH 6.8.
	*Additional components* (*filter sterilized with a syringe filter [0.22 μM, Merck]*)*:* 0.5% egg yolk emulsion (Sigma-Aldrich); 1% BSA (bovine serum albumin, VWR); 0.06% 1M MgCl_2_; 0.1% 3.6 mM FeSO_4_ × 7H_2_O; 0.175% 1 M CaCl_2_; 1–1% amino acid stock solutions I–VIII.
	*Stock solutions of amino acids (AA) (pre-prepared, filter-sterilized and stored at 4°C until use):* AA solution I: 53 mM L-phenylalanine, 212.8 mM L-lysine × HCl, 30.6 mM L-arginine × HCl.
	AA solution II: 120.3 mM L-glycine, 111.7 mM L-valine, 178 mM L-alanine, 1.3 mM L-tryptophan.
	AA solution III: 107.2 mM L-threonine, 144.6 mM L-serine, 166.1 mM L-proline.
	AA solution IV: 82.7 mM L-aspartate, 16 mM L-cysteine × HCl, 9.1% (v/v) 36.5% HCl.
	AA solution V: 154.9 mM L-glutamate × HCl, 80.2 mM L-tyrosine, 4% NaOH.
	AA solution VI: 160.9 mM L-leucine.
	AA solution VII: 67.6 mM L-ornithine × HCl, 63.3 mM L-methionine, 51.9 mM L-histidine × HCl.
	AA solution VIII: 112 mM L-isoleucine.
RPMI-1640	RPMI-1640 medium (Sigma-Aldrich) buffered with 0.165 M MOPS and supplemented with 0.03% L-glutamine
RPMI-1640 agar	RPMI-1640 supplemented 2% agar
MM	2.5% agar; 0.05% MgSO_4_; 0.05% KCl; 0.05% K_2_HPO_4_; 0.001% ZnSO_4_; 0.001% FeSO_4_; 0.0003% CuSO_4_, 0.3% NaNO_3_, 3% sucrose.
Carbon-depleted MM	MM without sucrose.
Nitrogen-depleted MM	MM without NaNO_3_.
Sulfur-depleted MM	2.5% agar; 0.05% MgCl_2_; 0.05% KCl; 0.05% K_2_HPO_4_; 0.001% ZnCl_2_, 0.001% FeCl_3_, 0.0003% CuCl_2_; 0.3% NaNO_3_; 3% sucrose.
Carbon- and nitrogen-depleted MM	MM without sucrose and NaNO_3_.
Carbon- and sulfur-depleted MM	Sulfur-depleted MM without sucrose.
Nitrogen- and sulfur-depleted MM	Sulfur-depleted MM without NaNO_3_.
Carbon-, nitrogen- and sulfur-depleted MM	Sulfur-depleted MM without sucrose and NaNO_3_.

*LB, lysogeny broth; MEA, malt extract agar; MM, minimal medium; RPMI, Roswell Park Memorial Institute; SCFM, synthetic cystic fibrosis medium.*

### *Pseudomonas aeruginosa* Cell Fractions

Heat killed and lysed bacterial cells and bacterial culture supernatants were prepared according to Pethani (2015) with slight modifications. In order to prepare heat killed cells, 1 ml of the 48 h culture of *P. aeruginosa* (0.5 × 10^9^ – 1.0 × 10^9^ CFU/ml) was heat treated at 80°C for 60 min. Ten microliters of the cultures were plated on solid LB medium and incubated overnight at 37°C to check the efficiency of the treatment. Cell culture supernatants were collected by centrifuging 1 ml of the 48 h cultures (0.5 × 10^9^ – 1.0 × 10^9^ CFU/ml) of *P. aeruginosa* strains at 10,000 × *g* for 30 min. The obtained supernatants were filtered through a 0.22 μm syringe filter. To prepare cell lysates, the remaining pellet was resuspended in 1 ml distilled water, chilled at −20°C, then sonicated for 30 min in an ultrasonic processor. The sonicated suspensions were centrifuged at 10,000 × *g* for 30 min to remove the cellular debris and the supernatant was filtered through a syringe filter with a pore size of 0.22 μm to remove the remaining cellular particles.

### Direct Interactions

Direct interactions between living fungal and bacterial strains were tested on synthetic cystic fibrosis medium (SCFM, Kozlowska et al., 2013) and on RPMI-1640 agar using the disk inhibition method. The compositions of these media are listed in Table 2. Firstly, the inhibitory effect of the fungal strains was tested against the bacteria. 100 μl of 10^4^ CFU/ml conidial suspensions were spread on the plates. After drying, sterile filter paper disks with a diameter of 0.8 mm were placed at the center of each plate and inoculated with 10 μl of 5 × 10^5^ CFU/ml *P. aeruginosa* suspension. As a control, the same amounts of bacterial cells were grown on sterile media. As an inverse of this test, inhibitory activity of the bacterial strains on the fungal growth was tested as follows: 100 μl of 10^5^ CFU/ml *P. aeruginosa* suspensions were spread onto the plates and 10 μl of 10^4^ CFU/ml conidial suspensions were added to the filter paper disks. The control plates were free of the *P. aeruginosa* cells. Inhibition percentages were calculated at 3 days post inoculation (dpi) by comparing the diameter of the central colonies grown on the co-culture plates to the control ones growing in mono-culture.

The effects of heat killed and lysed bacterial cells and bacterial culture supernatants on fungal growth were tested similarly by adding 10 or 50 μl of each cell fraction on the filter paper disk instead of a living cell suspension. All plates were incubated at 37°C for 3 days and the diameter of the inhibition zone was measured.

### Indirect Interactions via Volatile Organic Compounds (VOCs)

To examine indirect interactions via VOCs between fungi and bacteria, the so-called “plate-in-plate” method was used (Briard et al., 2016). This approach allows to grow bacteria and fungi in the same atmosphere but on different culture media, physically separately from each other.

In these experiments, 10 μl of 10^4^ CFU/ml fungal conidial suspensions were inoculated at the center of the inner plate, while 100 μl of 10^5^ CFU/ml bacterial suspensions were spread on the outer plate. Petri dishes were sealed with parafilm and placed in self lock plastic bags to prevent the VOCs from escaping into the incubator. After 7 days of incubation, the diameter of the fungal colonies was measured. Then, the entire colony was scraped carefully from the surface of the media using a sterile scalpel and dried in an oven at 70°C for 16 h to determine their dry weight. Lastly, biomass density (BD) values were calculated using the following formula of Reeslev and Kjoller (1995):

$$\text{BD}=\mathrm{dry weight}\;(\text{mg})/\mathrm{area of the colony}\;({\text{cm}}^{2}).$$

We started these experiments using nutrient rich media, such as RPMI-1640 agar and SCFM, for the cultivation of bacteria and fungi, as well. In the next eight experimental conditions, bacteria were grown uniformly on solid LB media to provide a stable environment for them through the tests and only the contents of the inner plate were changed. Based on the work of Briard et al. (2016) and the facts that VOCs are all carbon-based molecules and *P. aeruginosa* is able to produce both sulfur- and nitrogen-containing molecules along with a large variety of volatile metabolites (Bos et al., 2013), fungal isolates were grown on various nutrient-depleted media, i.e., minimal medium (MM), carbon-depleted MM, nitrogen- depleted MM, sulfur-depleted MM, and their combinations carbon- and nitrogen-depleted MM, carbon- and sulfur-depleted MM, nitrogen- and sulfur-depleted MM and finally carbon-, nitrogen- and sulfur-depleted MM. Compositions of these media are detailed in Table 2.

### Indirect Interactions via Media-Soluble Molecules

Indirect interactions via media-soluble molecules were investigated by the modified cellophane assay of Heatley (1947) on solid SCFM and RPMI-1640 media and by culturing the fungi in liquid RPMI-1640 conditioned by *P. aeruginosa* strains.

For the cellophane assay, the surface of the media was covered with a sterile cellophane sheet with the shape and size of a 90 mm Petri dish. Then a sterile filter paper disk with a diameter of 0.8 mm was placed at the center of the plate and inoculated with 10 μl of 5 × 10^5^ CFU/ml *P. aeruginosa* suspension, except for the control plates, which were inoculated with sterile distilled water. All the plates were incubated at 37°C. Before the cellophane membranes were uncovered from the plates along with the bacterial colonies at 2 dpi, colony diameters of *P. aeruginosa* were determined in two perpendicular directions. Then 100 μl of 2 × 10^4^ CFU/ml conidial suspensions were spread on the underlying medium. The diameters of inhibition zones indicating the penetration of bacterial small diffusible molecules through the cellophane membrane were determined after 3 days of incubation at 37°C. The area of colonies and inhibition zones were determined using the formula π × *a* × *b*, where “*a*” and “*b*” were the two radii measured in two perpendicular directions. Finally, to calculate the relative inhibition activity (RIA) of the bacteria, the area of each inhibition zone detected for the fungal colonies was divided by the area of the related bacterial colony.

To prepare conditioned media, 1-day-old cultures of *P. aeruginosa* strains grown in LB media were diluted to a density of 1 × 10^5^ CFU/ml in saline solution. Then, 10 ml of RPMI-1640 was inoculated with 100 μl of the diluted bacterial suspensions to reach the final density of 10^3^ CFU/ml and incubated at 37°C without shaking. Medium used for the growth control wells was inoculated with 100 μl of sterile saline solution and kept at 37°C, as well. At 2 dpi, both infected (containing 1.0 × 10^8^ – 5.0 × 10^8^ CFU/ml) and non-infected media were centrifuged at 4,000 rpm for 10 min at 15°C and the supernatants were filtered through a 0.45 μm and subsequently a 0.22 μm syringe filter (Merck). Then, pH of the conditioned and the control media were measured in order to confirm that bacteria do not affect the fungal growth by altering the neutrality of the media. The effect of the obtained sterile bacterial supernatants on the growth of filamentous fungal isolates was tested in 96-well microplates. One hundred microliters of the supernatant was mixed in each well with 100 μl conidial suspensions diluted in RPMI-1640 to a density of 2 × 10^4^ CFU/ml. Growth control wells contained 100 μl of uninfected but centrifuged and filter-sterilized RPMI-1640 mixed with 100 μl conidial suspensions. Sterile controls contained 200 μl treated and untreated media without conidia. Fungal growth was evaluated after 24, 48, and 72 h of incubation at 37°C by measuring the optical density of each sample at 620 nm with an absorbance plate reader (SPECTROstar Nano, BMG Labtech). The absorbances of the untreated control cultures were referred to 100% of growth in each case. Experiments were performed in three independent experiments with three individual replicates.

### *In vitro* Effect of Bacterial Signal Molecules, Antibacterial Agents, and Corticosteroids on Scedosporia

Two commercially available bacterial signal molecules, pyocyanin (Sigma-Aldrich) and diffusible signal factor (DSF; *cis*-11-methyl-2-dodecenoic acid; Sigma-Aldrich), three commonly used antibacterial agents (i.e., tobramycin, ceftazidime and flucloxacillin; Sigma-Aldrich) and three corticosteroids (i.e., hydrocortisone, prednisone, and methylprednisolone; Sigma-Aldrich) were tested in this experiment. The long-term (24–72 h) effect of these compounds were tested on all the 11 isolates by comparing the absorbance of the treated and non-treated samples using a plate reader as it was described in the previous subsection. Additionally, the short-term (2–8 h) effect of DSF and pyocyanin was also determined by investigating the germination ability of fungal conidia in the presence of these compounds under a light microscope: 20 fields of view (at least 100 conidia) were selected randomly from each sample where germinated and non-germinated conidia were counted. In these test, one representative clinical isolate was involved for each species, namely, *S. angustum* CBS 254.72, *S. boydii* CBS 117432, *S. ellipsoideum* CBS 301.79, *S. aurantiacum* CBS 136046, *S. apiospermum* SZMC 23374 and *A. fumigatus* SZMC 23245. Experiments were performed in three independent experiments with three individual replicates.

The test concentrations of each compound were selected based on the related literature. Where the reported (plasma and/or sputum) levels of a compound proved to be highly variable, the effect of more than one concentration was examined in the growth experiments. Accordingly, 12.5, 10, and 50 μg/ml of pyocyanin (Wilson et al., 1988), 50 μg/ml of DSF (Wang et al., 2004), 10 μg/ml flucloxacillin (Sutherland et al., 1970; Bodey et al., 1972), 10, 20, and 80 μg/ml of ceftazidime (Vinks et al., 1997; Moriarty et al., 2007; Hubert et al., 2009), 5, 10, and 20 μg/ml of tobramycin (Moriarty et al., 2007; VandenBussche and Homnick, 2012), 10^−6^ M (∼0.36 μg/ml) of corticosteroids (Ng et al., 1994) were used in these experiments. While the germination studies were conducted using the highest abovementioned values (50 μg/ml) of DSF and pyocyanin.

### Statistical Analyses

All statistical analyses were performed using GraphPad Prism 6 software. Unpaired *t*-test was used to reveal significant differences between the investigated groups. *P*-values < 0.05 were considered as statistically significant.

## Results

### *Pseudomonas aeruginosa* and *Scedosporium* Strains Exert a Contact-Dependent Inhibitory Effect on Each Other

**FIGURE 1:**
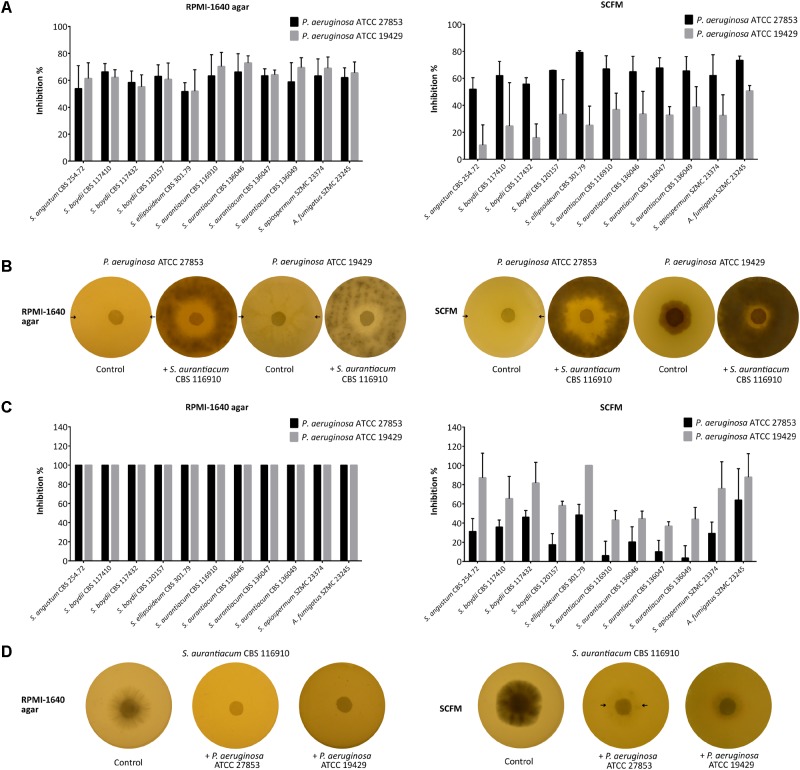
Direct interactions between *Pseudomonas aeruginosa* and *Scedosporium* strains tested by the disk inhibition method. In panel **(A)**, the graphs represent the mean inhibition percentages of the fungal isolates against *P. aeruginosa* ATCC 27853 and ATCC 19429 on RPMI-1640 agar **(left)** and on SCFM plates **(right)**. Error bars indicate standard deviations. Panel **(B)** is a representative figure of the plates after 3 days of co-incubation illustrating the direct inhibitory effect of *S. aurantiacum* CBS 116910 on the growth of *P. aeruginosa* ATCC 27853 and ATCC 19429 exerted on RPMI-1640 agar **(left)** and SCFM plates **(right)**. In panel **(C)**, the graphs represent the mean inhibition percentages of *P. aeruginosa* ATCC 27853 and ATCC 19429 against all fungal isolates on RPMI-1640 agar **(left)** and SCFM plates **(right)**. Error bars indicate standard deviations. Panel **(D)** is a representative figure of the plates after 3 days of co-incubation illustrating the direct inhibitory effect of *P. aeruginosa* ATCC 27853 and ATCC 19429 on the growth of *S. aurantiacum* CBS 116910 on RPMI-1640 agar **(left)** and SCFM plates **(right)**. Arrows indicate the borders of the colonies in case they are hardly visible.

Interactions between living bacterial and fungal cells were investigated using the disk inhibition test. These experiments revealed medium-specific interactions between the investigated microorganisms (Figure 1). On RPMI-1640 agar, the fungal strains acted similarly on *P. aeruginosa* having an approximately 60% inhibitory effect on both strains (Figure 1A,B, **left**). In the reverse case, both bacterial strains showed an equally strong inhibitory action (100%) on all the tested fungal isolates (Figure 1C,D, **left**). In comparison, on SCFM, scedosporia had a stronger inhibitory effect on *P. aeruginosa* ATCC 19429 (mean inhibitory percentage: 65.1 ± 7.4%) than on *P. aeruginosa* ATCC 27853 (mean inhibitory percentage: 47.7 ± 20.0%) (Figure 1A,B, **right**). Accordingly, *P. aeruginosa* ATCC 19429 had a less prominent antifungal effect on *Scedosporium* spp. than *P. aeruginosa* ATCC 27853 (Figure 1C,D, **right**). Additionally, on SCFM plates, both bacterial strains caused a less prominent inhibition of the four *S. aurantiacum* strains (ATCC 27853: 10.1 ± 7.3%; ATCC 19429: 42.1 ± 3.5%) than the other fungal species (ATCC 27853: 38.9 ± 15.2%; ATCC 19429: 79.5 ± 14.2%) (Figure 1C, **right**). Prominent differences were not observed among the bacterial growth inhibitory effect of the fungal strains (Figure 1A).

Interaction tests with living fungal and heat-killed bacterial cells were also performed. According to our results, only living bacterial cells were able to inhibit the fungal growth, and heat killed cells had no visible effect on the growth of scedosporia (data not shown).

### Bacterial Media-Soluble Molecules Possess Fungal Growth Inhibitory Activity

**FIGURE 2:**
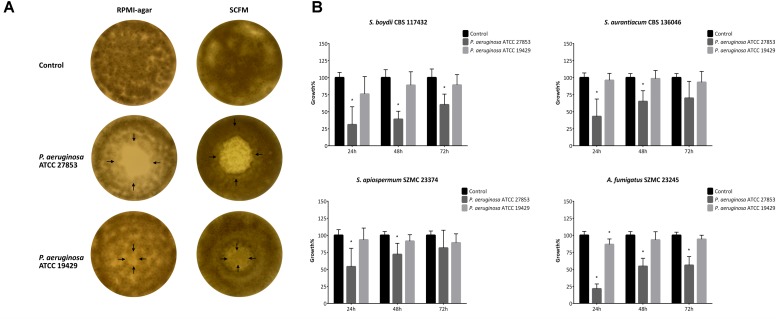
Bacterial media-soluble/diffusible molecules inhibit the growth of filamentous fungi both on solid **(A)** and in liquid **(B)** media. Panel **(A)** is a representative figure showing the influence of the bacterial diffusible molecules on the fungal lawn of *S. apiospermum* SZMC 23374 grown on RPMI-1640 and SCFM plates. Arrows indicate the borders of the bacterial colony determined in two perpendicular directions. In panel **(B)**, the graphs show the fungal growth rates in the presence of bacterial diffusible molecules in liquid culture media. Results are the mean from three independent experiments involving three individual replicates. Error bars indicate standard deviations. *P*-values were calculated using the unpaired *t*-test, where *p* < 0.05 was considered to be significant (indicated with asterisks). Graphs illustrating the growth rate of the other fungal strains tested are available in Supplementary Figure 1S.

The effect of bacterial diffusible molecules on fungal growth was tested on solid and in liquid culture media, as well. *P. aeruginosa* ATCC 27853 showed stronger indirect inhibitory effect in these tests than ATCC 19429 irrespectively to the type of media (solid or liquid) used. On solid RPMI-1640 plates, mainly complete inhibition zones, while on SCFM, only partial inhibition zones were observed at the former sites of the bacterial colonies (Figure 2A and Supplementary Table 1S). Similarly, in liquid RPMI-1640, the growth of the fungal isolates was unambiguously higher in the control samples, than in the medium conditioned by *P. aeruginosa* ATCC 27853. However, this antifungal activity decreased over time and was hardly detectable in case of the medium conditioned by *P. aeruginosa* ATCC 19429 (Figure 2B and Supplementary Figure 1S). All data obtained in the cellophane assay and the microdilution tests with conditioned media are available in the Supplementary Table 1S and Supplementary Figure 1S. According to our measurements, the pH of media conditioned by *P. aeruginosa* ATCC 27853 and ATCC 19429 were between 6.94 and 7.05, while that of the control media was in the range of 6.98–7.05. These results confirmed that *P. aeruginosa* did not affect the fungal growth through altering the pH of the media.

Effect of the bacterial culture supernatant and the cell lysate was also tested on solid RPMI-1640 and SCFM plates but none of them showed any visible fungal growth inhibitory effect in the disk inhibition tests (data not shown).

### Bacterial Signal Molecules Affect the Germination Ability and the Growth Rate of Scedosporia

**FIGURE 3:**
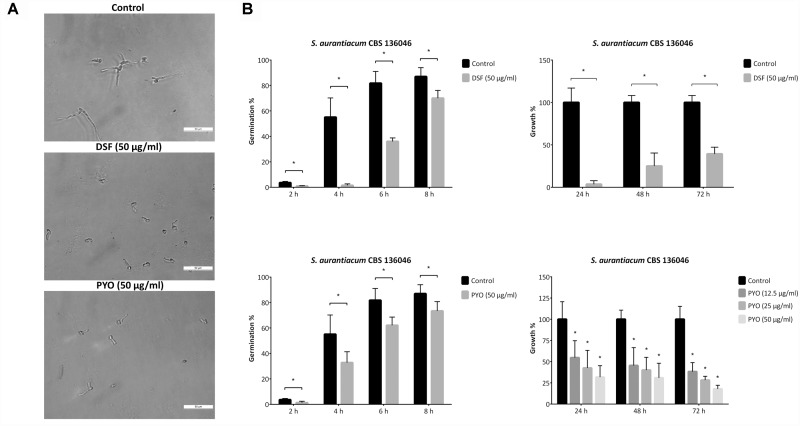
Diffusible signal factor (DSF) and pyocyanin (PYO) shows growth inhibitory effect against *S. aurantiacum*. Panel **(A)** shows the germ tube formation and elongation of *S. aurantiacum* CBS 136046 conidia after 8 h of co-incubation with 50 μg/ml of DSF or pyocyanin and in the control, DSF- and pyocyanin-free medium. Scale bar = 50 μm. Panel **(B)** shows the germination **(left panel)** and the growth rates **(right panel)** of *S. aurantiacum* CBS 136046 monitored over the indicated time course via microscopic and spectrophotometric analyses, respectively. The graphs show mean values with error bars indicating the standard deviations of three independent experiments. *P*-values were calculated using the unpaired *t*-test where *p* < 0.05 was considered to be significant (indicated with asterisks). Graphs illustrating the germination and growth rate of the other fungal strains tested are available in the Supplementary Figures 2S–5S.

The observed indirect antifungal activity of *P. aeruginosa* might be attributed to bacterial signal molecules. In order to examine this hypothesis, the effect of two known signal molecules, namely DSF and pyocyanin were tested on the germination and growth rate of scedosporia. These compounds were chosen based on their previously reported role in inter-kingdom interactions (Boon et al., 2008; Briard et al., 2015). Figure 3 illustrates our main findings on the clinical strain *S. aurantiacum* CBS 136046, the remaining results of the tests are available in Supplementary Figures 2S–5S. As a short-term effect, both DSF and pyocyanin were able to decrease the germination rate of scedosporia (Figure 3, **left panel**). Their long-term effect proved to be similar: the growth rate of fungi significantly decreased in the presence of these compounds after 24, 48, and 72 h of incubation (Figure 3, **right panel**). However, 50 μg/ml of DSF had a more prominent inhibitory effect on the germ tube formation and elongation than the same level of pyocyanin.

### Pseudomonal Nitrogen- and Sulfur-Containing VOCs Stimulate the Growth of *Scedosporium* spp.

**FIGURE 4:**
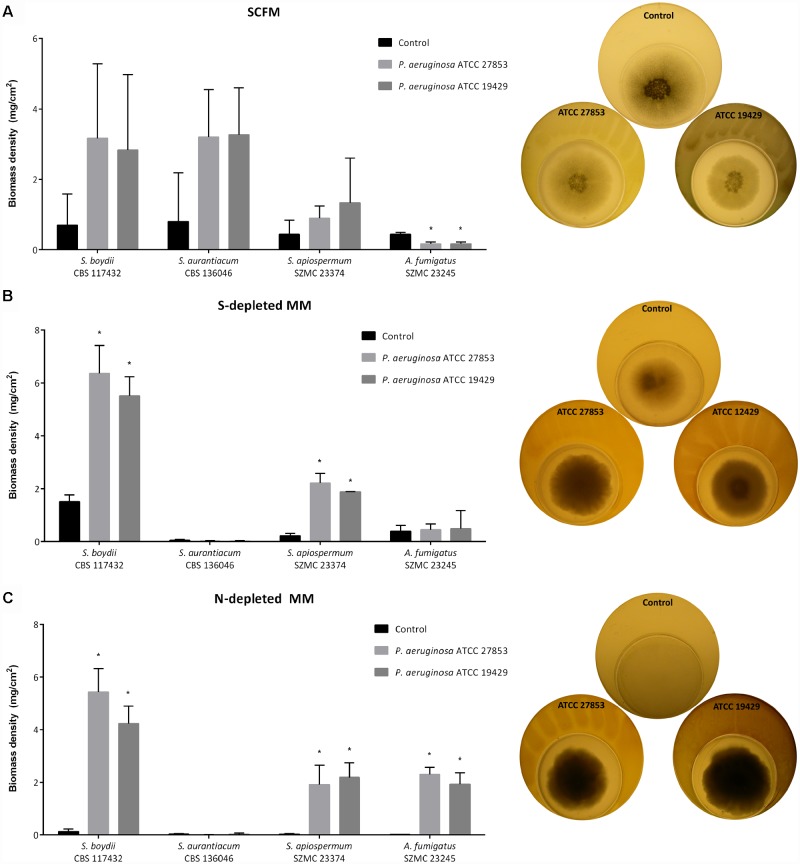
Representative results of the plate-in-plate (PIP) co-culture tests. The impact of bacterial VOCs on the growth of certain *Scedosporium* strains and *A. fumigatus* after 7 days of co-incubation at 37°C on SCFM **(A, left panel)**, on S-depleted MM **(B, left panel)** and on N-depleted MM; **(C, left panel)** are presented. *P*-values were calculated using the unpaired *t*-test, where *p* < 0.05 was considered as significant (indicated with asterisks). Graphs illustrating biomass densities of the other fungal strains and the results obtained on the other eight culture media are available in the Supplementary Figures 6S–15S. The right panels demonstrate the growth of *A. fumigatus* SZMC 23245 on SCFM **(A, right panel)** and *S. apiospermum* SZMC 23374 cultured on S- **(B, right panel)** and on N-depleted MM **(C, right panel)** in the presence or absence of bacteria.

The impact of bacterial VOCs on fungal growth were tested in plate-in-plates tests. Although the two pathogens were cultured physically separately from each other in these tests, the presence of bacteria could stimulate the growth of the fungi (Figure 4). This trend proved to be statistically significant for *S. angustum* CBS 254.72 and *A. fumigatus* SZMC 23245 isolates on RPMI-agar, and for *S. angustum* CBS 254.72 and *S. boydii* CBS 117410 strains on SCFM plates (Supplementary Figures 6S, 7S). Interestingly, the growth of *A. fumigatus* was not enhanced, but impaired by the VOCs of *P. aeruginosa* on SCFM (Figure 4A and Supplementary Figure 6S), suggesting that these interactions are not just strain- and species-dependent, but are also affected by the nutrients (un)available in the environment. It is also noteworthy that the biomass of *Scedosporium* spp. was lower on RPMI-1640 agar (median biomass density: 0.13 mg/cm^2^) than on SCFM plates (median biomass density: 0.5 mg/cm^2^) and, except for the abovementioned two cases, bacterial VOCs had no effect on fungal growth on this medium, at all.

To make a proposal for the groups of the volatiles responsible for these effects, fungal isolates were cultured on various nutrient-depleted media. On nitrogen-depleted MM, fungal growth proved to be very weak, the biomass densities of *Scedosporium* spp. ranged between 0.02 and 0.13 mg/cm^2^, with a median value of 0.03 mg/cm^2^. However, the presence of the bacteria enabled the growth of all *Scedosporium* isolates (median biomass density: 2.2 mg/cm^2^; biomass density range: 2.18–5.44 mg/cm^2^) with the exception of the *S. aurantiacum* strains (Figure 4C and Supplementary Figure 8S). On the sulfur-depleted medium, a similar scenario was observed; the four *S. aurantiacum* strains were not able to grow, even in the presence of the *P. aeruginosa* strains (biomass density ≤ 0.05 mg/cm^2^). While, the biomass densities of the *S. angustum*, *S. boydii*, *S. ellipsoideum*, and *S. apiospermum* strains were elevated (median: 2.15 mg/cm^2^; range: 1.65–6.36 mg/cm^2^) in the presence of the bacteria, compared to the bacterium-free, control plates (median: 0.26 mg/cm^2^; range: 0.1–1.51 mg/cm^2^) (Figure 4B and Supplementary Figure 9S). Similar results were obtained on nitrogen- and sulfur-depleted MM and also on MM containing only inorganic salts and sucrose (Supplementary Figures 10S, 11S). Contrastingly, without any carbon source in the medium all the isolates grew sparsely (biomass density < 0.05 mg/cm^2^) and even the presence of bacteria could not stimulate their growth significantly (Supplementary Figure 13S). In case of carbon- and nitrogen-depletion, only the growth of *A. fumigatus* was enhanced significantly by the presence of the *Pseudomonas* strains (Supplementary Figure 12S). Under simultaneous carbon and sulfur starvation, the presence of the bacteria had neither negative, nor positive impact on the growth of fungal isolates (Supplementary Figure 14S). Finally, on carbon-, nitrogen-, and sulfur-depleted MM, the strain *P. aeruginosa* ATCC 192429 had a significant growth inhibitory effect on two *S. boydii* and two *S. aurantiacum* isolates, whereas the biomass of *A. fumigatus* proved to be significantly increased in its presence (Supplementary Figure 15S). In summary, the significant growth-promoting effect *P. aeruginosa* was detected on nitrogen- and/or sulfur-depleted MM, suggesting that volatile nitrogen- and sulfur-containing compounds might be in the background of the growth stimulating effect.

The graphs on Figure 4B,C also shows that the representatives of *S. aurantiacum* – unlike *S. boydii* or *S. apiospermum* isolates – were not able to utilize the bacterial nitrogen- and sulfur-containing VOCs from the air, but required these chemical elements fixed in the media for their growth. This observation suggests some fundamental differences between the nitrogen and sulfur metabolism of *S. aurantiacum* and the other tested *Scedosporium* species and even *A. fumigatus*.

### *In vitro* Effect of Corticosteroids and Antibacterial Agents on Scedosporia

**FIGURE 5:**
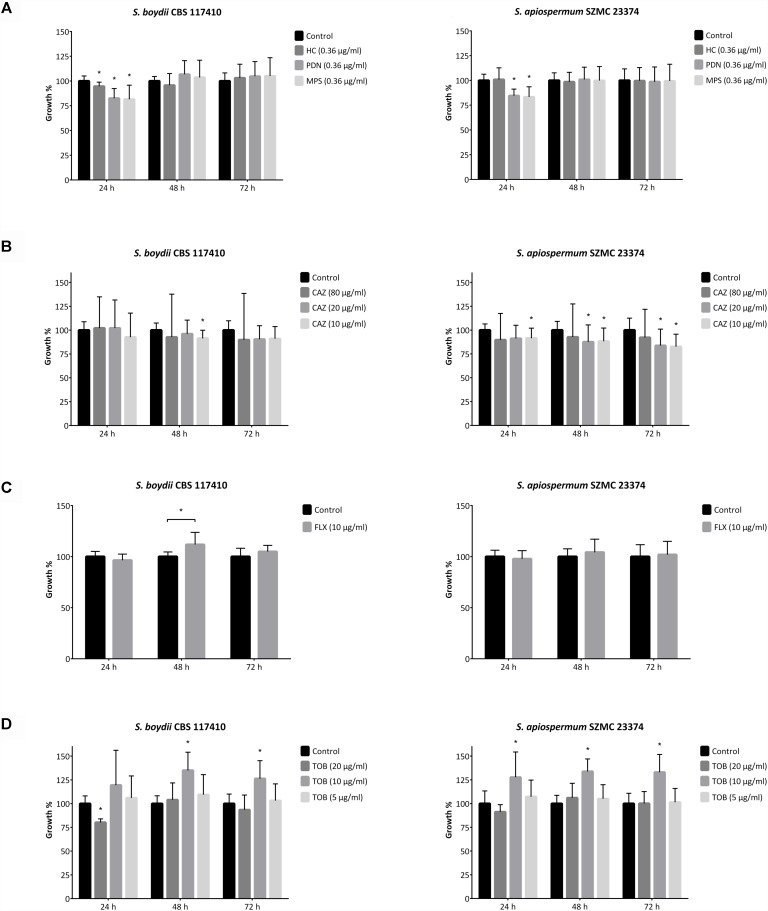
Direct fungal growth influencing effect of antibacterial agents and corticosteroids. Growth rate of *Scedosporium boydii* CBS 117410 and *S. apiospermum* SZMC 23374 in the presence of **(A)** 10^−6^ M (∼0.36 μg/ml) corticosteroid compounds (i.e., hydrocortisone, HC; prednisone, PDN and methylprednisolone, MPS), **(B)** ceftazidime (CAZ), **(C)** flucloxacillin (FLX) and **(D)** tobramycin (TOB). Results are the mean of three independent experiments with three individual replicates. Error bars indicate standard deviations. *P*-values were calculated using unpaired *t*-test, where *p* < 0.05 was considered to be significant (indicated with asterisks).

In order to examine the possible side effects of a corticosteroid therapy in CF patients, the effect of pharmacologically relevant concentration of corticosteroids was investigated on the growth of scedosporia. According to our results summarized in Figure 5 and Supplementary Figure 16S, the growth rate of the isolates remained generally unaffected by the presence of 10^−6^ M hydrocortisone. Although a small, but significant decrease was observed in case of the *S. angustum* and an *S. boydii* isolate. Furthermore, after 24 h of co-incubation with prednisone and methylprednisolone the growth of the vast majority of isolates proved to be lower compared to the steroid-free samples. In contrast, these compounds showed a significant growth promoting effect on *A. fumigatus* at the 48th hour of incubation.

Clinical studies showed that a prior antibacterial therapy (i.e., flucloxacillin and tobramycin) may increase the risk of fungal colonization and infections in CF (Burns et al., 1999; Blyth et al., 2010). To test the background of this phenomenon, the direct effect of three antibiotics usually taken by CF patients were tested against scedosporia (Hubert et al., 2009; Blyth et al., 2010; VandenBussche and Homnick, 2012). We observed that the presence of tobramycin in the culture media was able to increase the growth rate of the isolates (Figure 5 and Supplementary Figure 17S). These differences proved to be statistically significant when 10 μg/ml of tobramycin was applied, but interestingly neither 5 nor 20 μg/ml of the drug caused a similar prominent effect. In the presence of 10 and 20 μg/ml ceftazidime, *Scedosporium* and *A. fumigatus* isolates showed decreased growth properties (Figure 5 and Supplementary Figure 18S). When exposed to 80 μg/ml of the same antibacterial agent, the growth of fungi proved to be highly variable compared to the control samples. Finally, flucloxacillin influenced positively the growth of *S. boydii*, *S. ellipsoideum* and *A. fumigatus* isolates after 48 and/or 72 h of incubation, but it caused a significantly decreased growth in *S. angustum* after the first day of incubation (Figure 5 and Supplementary Figure 19S).

## Discussion

This comprehensive study focused on the *in vitro* interactions of *P. aeruginosa* and *Scedosporium* strains. The direct influence of commonly used antibacterial agents and corticosteroids on the growth of *Scedosporium* spp. was investigated, as well.

### *Pseudomonas aeruginosa* and *Scedosporium* Strains Exert a Contact-Dependent Inhibitory Effect on Each Other

In agreement with our data, the antifungal activity of *P. aeruginosa* was confirmed on solid media in direct contact with *S. aurantiacum* by Kaur et al. (2015), Pethani (2015), and Chen et al. (2018), too. These studies also proved that heat killed cells caused no visible fungal growth inhibition indicating the necessity of living *P. aeruginosa* cells to inhibit the growth of *Scedosporium* species (Pethani, 2015). According to Kaur et al. (2015) inhibition pathways might only be induced in bacterial-fungal co-cultures. In addition to these studies, our results suggest that in direct physical contact not just *P. aeruginosa* was able to inhibit the growth of *Scedosporium* spp., but these fungi were also capable to inhibit the bacterial growth. In contrast to this observation, conidia and cell fractions of *S. aurantiacum* failed to display any inhibition against *P. aeruginosa* previously (Kaur et al., 2015), which may be explained by the distinct experimental approach applied in this study. Similarly to us, Reece et al. (2018) observed a mutually antagonistic relationship between *P. aeruginosa* and *A. fumigatus*. This study identified gliotoxin as the main inhibitory agent of bacterial growth in the culture supernatant of *A. fumigatus* (Reece et al., 2018). To our knowledge, toxins have not been described from *Scedosporium* species before. Therefore, the background of their antagonistic effect against bacteria is yet to be investigated in the future.

### Bacterial Media-Soluble Molecules Possess Fungal Growth Inhibitory Activity

The results of the cellophane assay and the broth microdilution tests revealed that bacterial diffusible molecules might have fungal growth inhibitory effect on *Scedosporium* spp. Whereas, 10 or 50 μl of the bacterial cell culture supernatants had no effect in the disk inhibition assay. Prior to the present study, 100 μl of culture supernatants and cell lysates of *P. aeruginosa* were reported to possess inhibitory action on *S. aurantiacum* strains in agar diffusion tests (Pethani, 2015). Although, this seems to contrast with our results, it should be noted that, similarly to us, Pethani (2015) observed that 10 μl of the bacterial culture supernatant or the cell lysate did not cause fungal growth inhibition. This concentration dependent action of the bacterial supernatant is also supported by the fact that it caused significant inhibition in the microdilution tests where fungal conidia and the bacterial cell culture supernatant were mixed in a 1:1 ratio. Similarly, in the cellophane assay where the media under the bacterial colony might be corresponded with a bacterial culture supernatant, both bacterial strains were able to affect the growth of *Scedosporium* spp. causing complete or partial inhibition zones.

Besides, Pethani (2015) observed that environmental *S. aurantiacum* strains were more susceptible to bacterial supernatants than the clinical strains. We did not observe this phenomenon neither among *S. aurantiacum*, nor among the other *Scedosporium* strains tested. According to Mowat et al. (2010) extracellular, soluble and heat-stable factors might be in the background of the fungal growth inhibitory effect of the supernatant of *P. aeruginosa*. As it was previously reported, soluble metabolites are often responsible for short distance interactions (Tyc et al., 2017), which is in line with our findings, that inhibition zones did not exceed the boundaries of bacterial colonies in the cellophane assay.

### DSF and Pyocyanin Affect the Germination Ability and the Growth Rate of Scedosporia

According to the literature, the observed direct and indirect inhibitory effect of *P. aeruginosa* might be attributed to DSF family members and phenazines, e.g., pyocyanin (Wang et al., 2004; Boon et al., 2008; Briard et al., 2015). The DSF family includes structurally related, bacterial quorum sensing signal molecules, to be more precise, *cis*-2-unsaturated fatty acids of different chain length and branching patterns. These factors play an important role in virulence by regulating biofilm formation, antibiotic tolerance and might be involved in interspecies interactions (Jennings et al., 2012; Ryan et al., 2015). Additionally, *in vivo* studies revealed that DSF promoted the persistence of *P. aeruginosa* in the infected lung of CF mice and enhanced the antibacterial resistance of bacterial biofilms (Twomey et al., 2012). Furthermore, these compounds play a role in inter-kingdom interactions, too. DSF family molecules proved to inhibit the morphological transition in *Candida albicans*, however, with a varying efficiency (Boon et al., 2008). In DSF-free medium, more than 50% of the yeast cells germinated, in contrast to this, both 50 and 5 μg/ml of DSF decreased this number under 10% (Wang et al., 2004). Our findings confirmed these results in *Scedosporium* and *Aspergillus* isolates, we observed a significant decrease in their germination and growth rate in the presence of 50 μg/ml of DSF (Figure 3 and Supplementary Figures 3S, 4S).

Pyocyanin is a redox-active, blue colored phenazine pigment produced by *P. aeruginosa* under quorum sensing regulation. It is a major virulence factor of the species, which helps the pathogen in the adaptation to the host environment and causes a wide array of damages in the infected host cells (Denning et al., 1998; Usher et al., 2002; O’Malley et al., 2003, 2004; Winstanley and Fothergill, 2009). Pyocyanin may also mediate antagonistic interaction between *P. aeruginosa* and its bacterial or fungal competitors (e.g., *C. albicans*, *Aspergillus* spp.; Kerr et al., 1999). However, this effect is thought to be restricted to aerobic conditions, unlike the CF lung, which is commonly described as an oxygen-limited environment (Winstanley and Fothergill, 2009). According to Briard et al. (2015), phenazines of *P. aeruginosa* exerts their antifungal effect by inducing reactive oxygen and nitrogen species apart from 1-hydroxyphenazine, which acts as an iron chelator and induces iron starvation in fungi. This study also showed, that the sub-inhibitory concentrations of pyocyanin, phenazine-1-carboxamide and phenazine-1-carboxylic acid had growth-promoting effect on *A. fumigatus*, by reducing Fe(III) to the more soluble Fe(II), which is taken up by the fungus. In summary, phenazines possess a dose-dependent, dual effect on fungi, enabling finely regulated interactions between *P. aeruginosa* and *A. fumigatus*. The minimum inhibitory concentration (MIC) of pyocyanin against *A. fumigatus* was 2 mM (∼400 μg/ml; Briard et al., 2015). While in two additional studies, the MIC values ranged between 40 and 128 μg/ml against *Candida* spp. and *Cryptococcus neoformans* and proved to be 64 μg/ml against *A. fumigatus* and *A. flavus* isolates (Sudhakar and Karpagam, 2011; Sudhakar et al., 2013). In the present study, we focused on the previously measured peak levels of pyocyanin in the sputum (Wilson et al., 1988), which are generally lower than the MICs determined by previous reports. Thus, as it was expected based on the abovementioned studies, the ≤50 μg/ml levels of pyocyanin could not cause complete growth inhibition in *Scedosporium* and *A. fumigatus* isolates, but significantly decreased their growth rates in almost every case (Figure 3 and Supplementary Figures 5S, 6S). In contrast to this, Kaur et al. (2015) observed no inhibitory effect on *S. aurantiacum* strains using crude phenazine extracts with unknown concentrations. The authors hypothesized that *S. aurantiacum* might be able to modify and inactivate phenazine molecules through a detoxification mechanism. However, it is also possible that the pyocyanin concentration of their crude extract was out of the range investigated in the present study (<12.5 μg/ml). In contrast to our results, 10 mM (∼2.1 mg/ml) commercial pyocyanin had no effect on *S. aurantiacum* strains in the study of Kaur et al. (2015). This might be attributed to the differences of the techniques applied. While Kaur et al. (2015) used the disk inhibition assay on solid SCFM, we applied the broth microdilution test in RPMI-1640 (the density of SCFM does not allow its use in the broth microdilution assay).

### Pseudomonal Nitrogen- and Sulfur-Containing VOCs Stimulate the Growth of *Scedosporium* spp.

Microbial VOCs serve as important mediators of intra- and interspecies long-distance communication. These molecules enable the producers to repel or inhibit the competitor organisms and attract or enhance the growth of the mutualistic ones (Effmert et al., 2012; Schulz-Bohm et al., 2017). For instance, soil and plant-associated bacteria are reported to produce VOCs having antifungal activity on common phytopathogenic fungi (Cordero et al., 2014; Chaves-López et al., 2015; Ossowicki et al., 2017). Moreover, certain microbial VOCs proved to possess plant growth promoting effects, as well. Because of these favorable properties, VOC-producing microorganisms are considered to be promising biocontrol agents in the future (Schulz-Bohm et al., 2017). On the other hand, VOCs may have a connection to human health issues, as well. For instance, they are believed to be appropriate biomarkers of human microbial infections and serve as an alternative, non-invasive way for diagnosis (Lawal et al., 2018). Besides, it is also suspected that volatile compounds have an important role among the members of the normal human microflora and the (co)infecting microbes during pathogenesis, but this field is still not fully discovered. When Briard et al. (2016) investigated the *in vitro* interactions of the two most common CF pathogens, they revealed for the first time that *P. aeruginosa* and other Gram-negative bacteria (i.e., *Escherichia coli* and *Burkholderia cepacia*) was able to stimulate fungal growth from a distance. Similarly, the present study confirms firstly this fungal growth stimulatory effect of volatiles produced by *P. aeruginosa* on *Scedosporium* species.

Briard et al. (2016) identified dimethyl sulfide as the main VOC responsible for this effect and observed that another sulfur-containing VOC, dimethyl disulfide could also stimulate the growth of *A. fumigatus* in the lack of sulfur, but it had no growth stimulatory effects in sulfur-replete medium. Contrastingly, the sulfur-containing VOCs (i.e., sulfur-methyl thioacetate, dimethyl disulfide and dimethyl trisulfide) of another *Pseudomonas* species, applied either alone or in combination affected negatively the growth of phytopathogenic fungal strains (Ossowicki et al., 2017). Furthermore, Chaves-López et al. (2015) identified a volatile molecule produced by *Bacillus* sp., called 3-hydroxy-2-butanone, that possess both stimulatory and inhibitory effect depending on the targeted fungal species. Our study confirmed the results of Briard et al. (2016) on sulfur-depleted media and raised the possibility that beside sulfur, nitrogen-containing VOCs might also have growth stimulatory effect on scedosporia. To our knowledge, the effect of nitrogen-containing VOCs of *P. aeruginosa* was not investigated before on the growth of *Scedosporium* or *Aspergillus* species.

### *In vitro* Effect of Antibacterial Agents and Corticosteroids on Scedosporia

Our results regarding the direct fungal growth-influencing effect of corticosteroids seem to be controversial, especially considering the previous study of Ng et al. (1994), who exposed *Aspergillus* conidia to different doses of hydrocortisone and examined their germination rate. The authors detected the species-specific growth-promoting effect of this compound and reported 40% increase in the growth rate of *A. fumigatus* in the presence of 10^−6^ M hydrocortisone, while the growth of *A. flavus*, *A. niger* and *A. oryzae* proved to be unaffected by the same concentration. This latter observation might explain our results as well, namely that corticosteroids had an opposite effect on the growth of *Scedosporium* and *A. fumigatus* isolates. The results of Ng et al. (1994) have not yet been confirmed by clinical studies. Sudfeld et al. (2010) noted that inhaled corticosteroids surprisingly decreased the likelihood of a fungal infection, which is roughly in line with our *in vitro* data. While, Blyth et al. (2010) found no correlation between corticosteroid therapy and *Scedosporium* colonization.

Clinical studies revealed that a prior antibacterial therapy in CF patients might be a risk factor for fungal infections (Burns et al., 1999; Blyth et al., 2010). Nonetheless, this is the first study that investigated the direct effect of antibacterial agents on the growth of scedosporia. Our *in vitro* data are comparable to previous clinical studies. Sudfeld et al. (2010) suggested chronic oral antibiotics as one of the main risk factors for filamentous fungal infections of CF patients. In the same work, the administration of inhaled tobramycin did not increase the incidence of fungal isolation. These data suggest that tobramycin might possess a dose-dependent effect on fungi. We assume, that inhaled tobramycin results in a much higher drug level in the lung tissues or in the sputum of CF patients than the oral route, and these high drug concentrations might affect fungal growth negatively as it was previously demonstrated *in vitro* against *Fusarium* and *Aspergillus* spp., while its lower concentrations might possess fungal growth-promoting effect (Day et al., 2009; Shrestha et al., 2015). However, this hypothesis contradicts with the study of Burns et al. (1999), who observed an increased isolation of *C. albicans* and *Aspergillus* species in the group treated with inhaled tobramycin compared with the placebo group. Furthermore, Blyth et al. (2010) reported that antistaphylococcal penicillins, such as flucloxacillin increased the risk for *Scedosporium* colonization, but it was not affected significantly by aminoglycosides (e.g., tobramycin).

## Conclusion

As all *in vitro* tests do, our microbial interaction assays simplify the environmental conditions, and thus, cannot completely simulate all *in vivo* parameters, partly, because it is hard to reconstruct such a complex environment as a CF lung and partly, because probably not all the important factors are known by now (Kerr, 1999). For example, one of such factors can be the humidity, which may have indirect effect on fungal growth by influencing the functionality of the mucociliary clearance (Williams et al., 1996). Regarding the plate-in-plate tests, it is noteworthy that metabolomes are time-dependent and conditional. Furthermore, it might be strain-specific and can be modulated by the environmental conditions, e.g., nutrition availability, interactions with other species, etc. (Bean et al., 2016; Neerincx et al., 2016). Therefore, the real-life interactions between the investigated pathogens are probably far more complicated with the presence of the normal microbial communities and the wide range of host factors, as it was experienced in the *in vitro* experimental conditions we used in this study. That is why, we would like to consider *in vivo* methods to investigate the pathogen–pathogen interactions and to test the influence of a prior corticosteroid or antibacterial therapy (e.g., tobramycin or flucloxacillin) on *Scedosporium* infections in the future.

In conclusion, we confirmed that *P. aeruginosa* inhibited the fungal growth in direct contact with *Scedosporium* species and *A. fumigatus*. Besides, we observed that without any physical contact, *P. aeruginosa* was able to enhance the growth of scedosporia via VOCs. However, this growth-promoting effect turned out to be species-specific and depended on the availability of nutrients in the environment: major differences were observed between *S. aurantiacum* and the members of the *S. apiospermum* species complex on sulfur- and nitrogen-depleted media. Our results suggest, that both antibacterial agents and corticosteroids might affect the growth properties of filamentous fungi underlying the importance of a carefully selected therapy for patients with CF.

## Data Availability

All datasets generated for this study are included in the manuscript and/or the Supplementary Files.

## Author Contributions

TP and CV contributed to the design and implementation of the research, and participated in drafting the manuscript. GN and ET contributed to analyze the results and helped in drafting the manuscript. CS and ET helped in the microscopic and spectrophotometric analyses. MH and AS performed the interaction and the microdilution tests, conducted statistical analyses, designed the figures and the tables and drafted the manuscript. All authors read and approved the final manuscript.

## Conflict of Interest Statement

The authors declare that the research was conducted in the absence of any commercial or financial relationships that could be construed as a potential conflict of interest.
