# Supplementary material for: In vitro Interactions of Pseudomonas aeruginosa With Scedosporium Species Frequently Associated With Cystic Fibrosis
Source: Front Microbiol. 2019 Mar 6;10:441. doi: 10.3389/fmicb.2019.00441 (PMC6414507; doi:10.3389/fmicb.2019.00441)
Supplement: Supplementary file 1 [file Data_Sheet_1.pdf]

In Vitro Interactions of *Pseudomonas aeruginosa* with *Scedosporium* Species Frequently Associated with Cystic Fibrosis

Mónika Homa[1,2*], Alexandra Sándor[1,2], Eszter Tóth[1,3], Csilla Szebenyi[1,2,3], Gábor Nagy[1,3], Csaba Vágvölgyi[2,3], Tamás Papp[1,2,3]

[1]MTA-SZTE "Lendület" Fungal Pathogenicity Mechanisms Research Group, Szeged, Hungary
[2]Department of Microbiology, Faculty of Science and Informatics, University of Szeged, Szeged, Hungary
[3]Interdisciplinary Excellence Centre, Department of Microbiology, University of Szeged, Szeged, Hungary

***Correspondence:**
Mónika Homa
homamoni@gmail.com

Table 1S Indirect inhibitory effect of bacterial diffusible molecules on the growth of *Scedosporium* spp. and *A. fumigatus* SZMC 23245 grown on RPMI-agar and on SCFM plates. While on RPMI-1640 agar mainly complete, on SCFM only partial inhibition zones were observed. Partial inhibition was read at the first point of significant reduce in growth (approximately 50-80% as judged by the naked eye). Results are the mean of three independent experiments.

Strain	***P. aeruginosa*** **ATCC 27853**		***P. aeruginosa*** **ATCC 19429**	
	**Complete inhibition (RIA±SD)**	**Partial inhibition (RIA±SD)**	**Complete inhibition (RIA±SD)**	**Partial inhibition (RIA±SD)**
**RPMI-agar**				
S. angustum CBS 254.72	+ (0.16±0.14)	+ (0.40±0.00)	-	+ (0.71±0.48)
S. boydii CBS 117410	+ (0.38±0.16)	-	+ (0.70±0.48)	+ (0.08±0.17)
S. boydii CBS 117432	+ (0.83±0.13)	-	-	+ (0.50±0.00)
S. boydii CBS 120157	+ (0.17±0.19)	+ (0.72±0.39)	-	+ (0.49±0.41)
S. ellipsoideum CBS 301.79	+ (0.24±0.26)	-	-	+ (0.65±0.47)
S. aurantiacum CBS 116910	+ (0.20±0.22)	-	-	+ (0.33±0.58)
S. aurantiacum CBS 136046	+ (0.40±0.11)	-	-	+ (0.06±0.11)
S. aurantiacum CBS 136047	+ (0.17±0.24)	+ (0.10±0.00))	-	+ (0.25±0.50)
S. aurantiacum CBS 136049	+ (0.53±0.05)	-	-	+ (0.07±0.15)
S. apiospermum SZMC 23374	+ (0.28±0.17)	-	-	+ (0.17±0.26)
A. fumigatus SZMC 23245	+ (0.75±0.23)	-	-	-
**SCFM**				
S. angustum CBS 254.72	-	+ (0.09±0.17)	-	-
S. boydii CBS 117410	-	+ (0.23±0.26)	-	-
S. boydii CBS 117432	-	-	-	+ (0.61±0.54)
S. boydii CBS 120157	-	+ (0.32±0.28)	-	+ (0.33±0,58)
S. ellipsoideum CBS 301.79	-	+ (0.17±0,29)	-	+ (0.33±0,58)
S. aurantiacum CBS 116910	-	+ (0.21±0.19)	-	+ (0.33±0,58)
S. aurantiacum CBS 136046	-	+ (0.72±0.34)	-	+ (0.33±0,58)
S. aurantiacum CBS 136047	-	+ (0.39±0.09)	-	-
S. aurantiacum CBS 136049	-	+ (0.57±0,18)	-	-
S. apiospermum SZMC 23374	-	+ (0.46±0.10)	-	+ (1.00±0.87)
A. fumigatus SZMC 23245	-	-	-	-

+, inhibition zone was observed; -, inhibition zone was not detected;
Abbreviations: ATCC, American Type Culture Collection; **CBS**, Centraalbureau voor Schimmelcultures; **RIA**, relative inhibitory activity (RIA = area of the inhibition zone/area of the related bacterial colony); **SCFM**, synthetic cystic fibrosis medium; **SZMC**, Szeged Microbiological Collection.

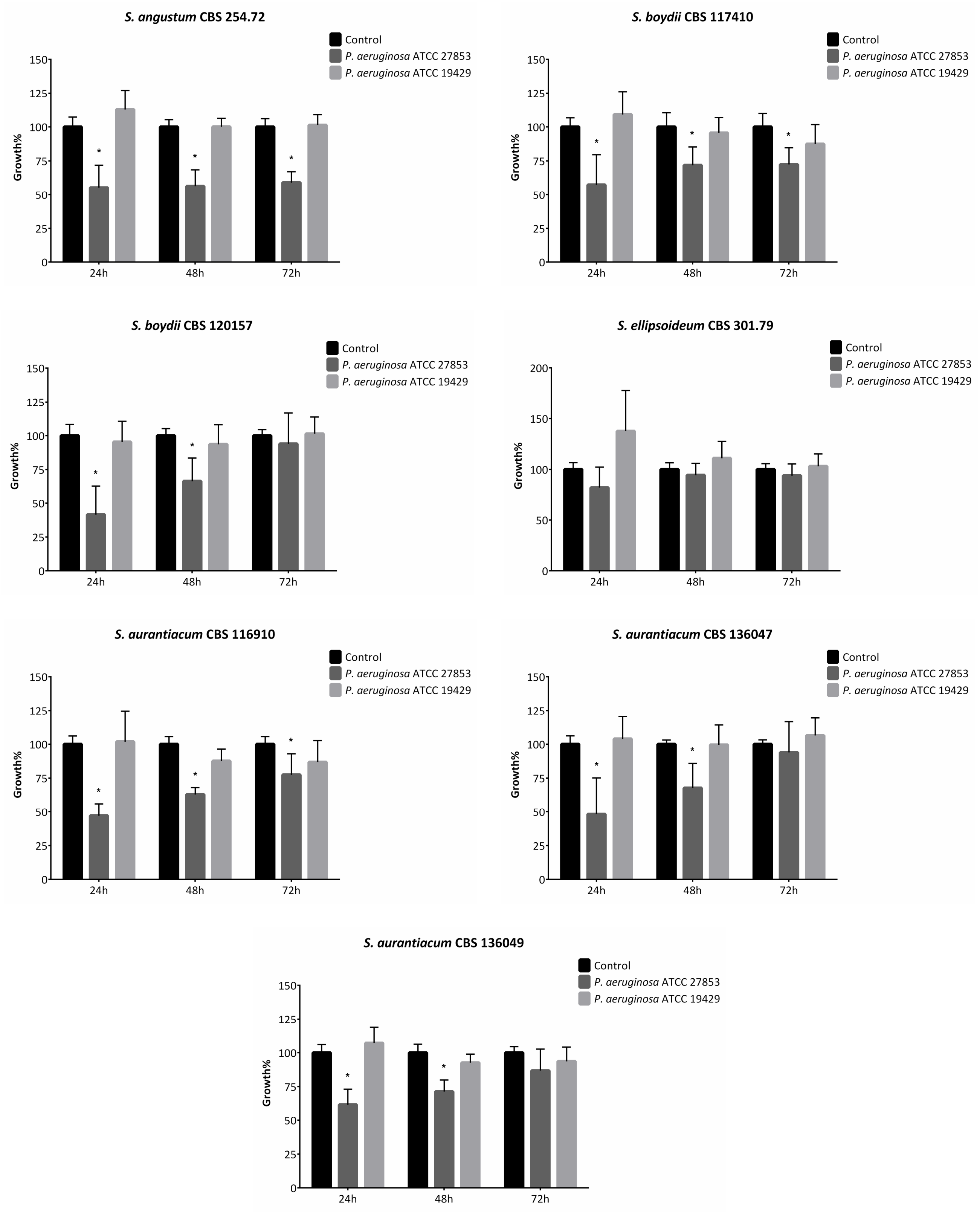

Figure 1S Indirect inhibitory effect of bacterial diffusible molecules on the growth of *Scedosporium* spp. grown in RPMI-1640 conditioned by *P. aeruginosa* strains. Results are the mean of three independent experiments with three individual replicates. Error bars indicate standard deviations. P values were calculated using unpaired t test, where $p<0.05$ was considered to be significant (indicated with asterisks).

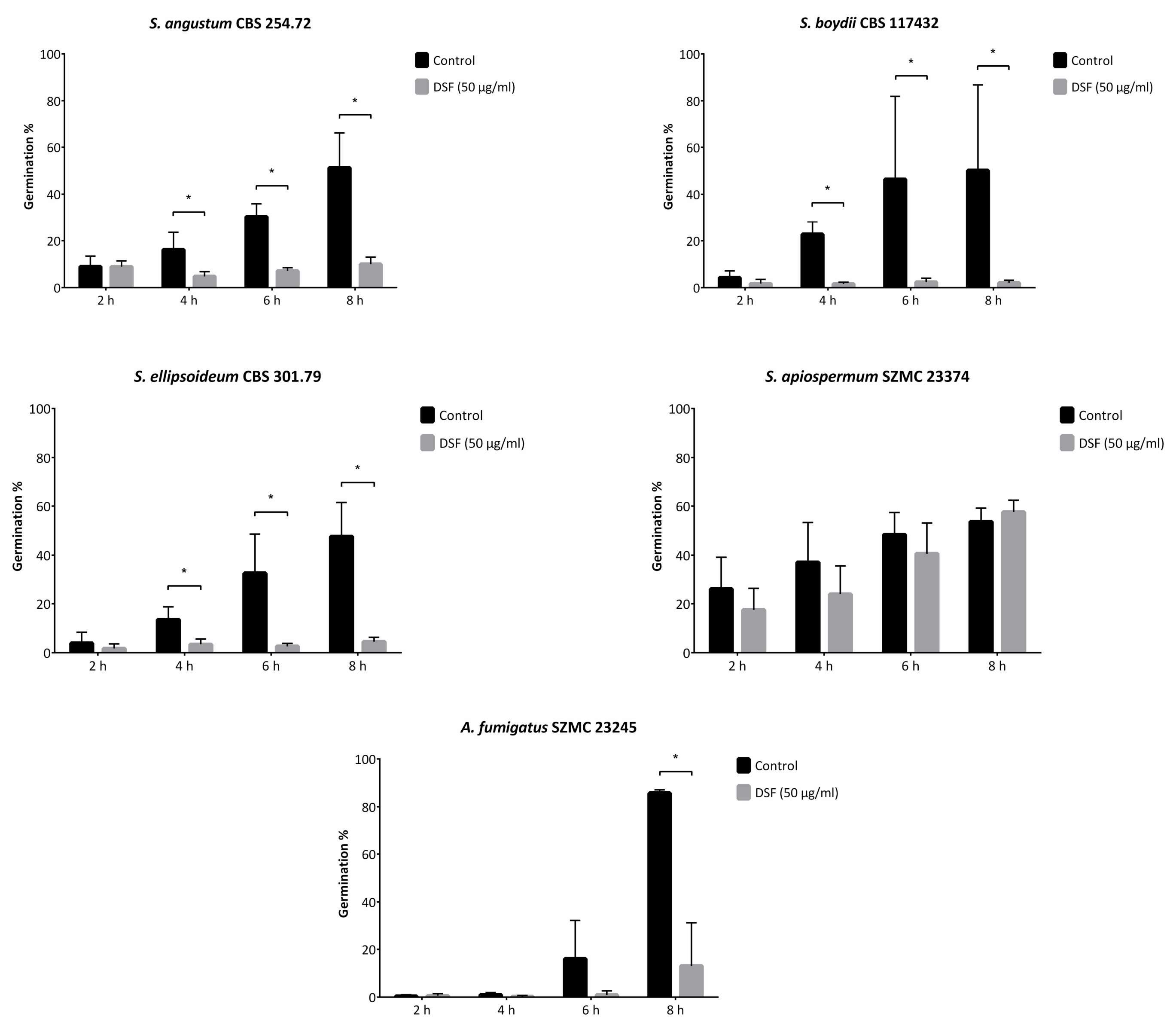

Figure 2S Germination ability of conidia collected from *Scedosporium* and *A. fumigatus* isolates in the presence of 50 µg/ml DSF. Results are the mean of three independent experiments. Error bars indicate standard deviations. P values were calculated using unpaired t test, where $p<0.05$ was considered to be significant (indicated with asterisks).

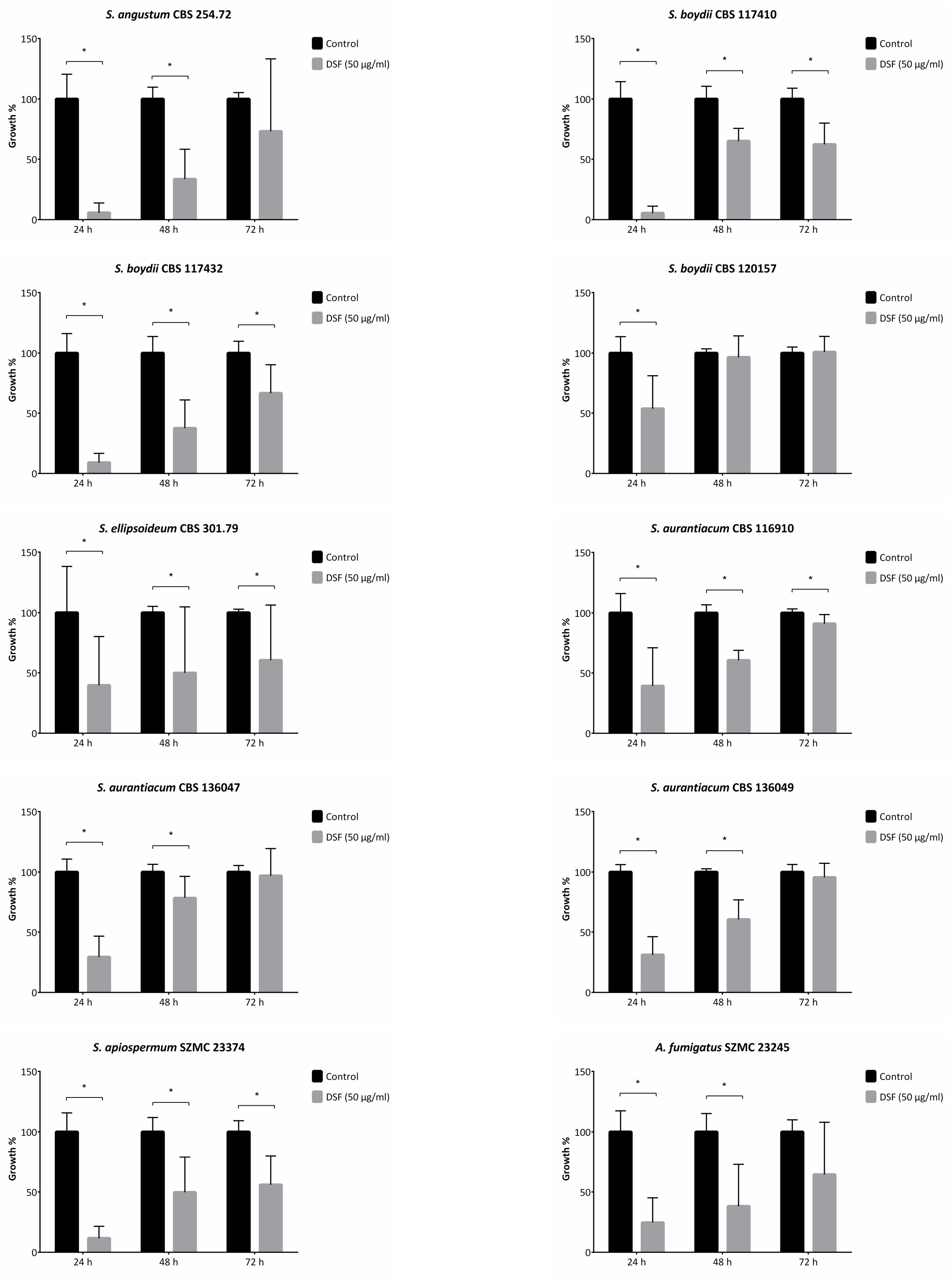

Figure 3S Growth rate of *Scedosporium* and *A. fumigatus* isolates in the presence of 50 µg/ml DSF. Results are the mean of three independent experiments with three individual replicates. Error bars indicate standard deviations. P values were calculated using unpaired t test, where $p<0.05$ was considered to be significant (indicated with asterisks).

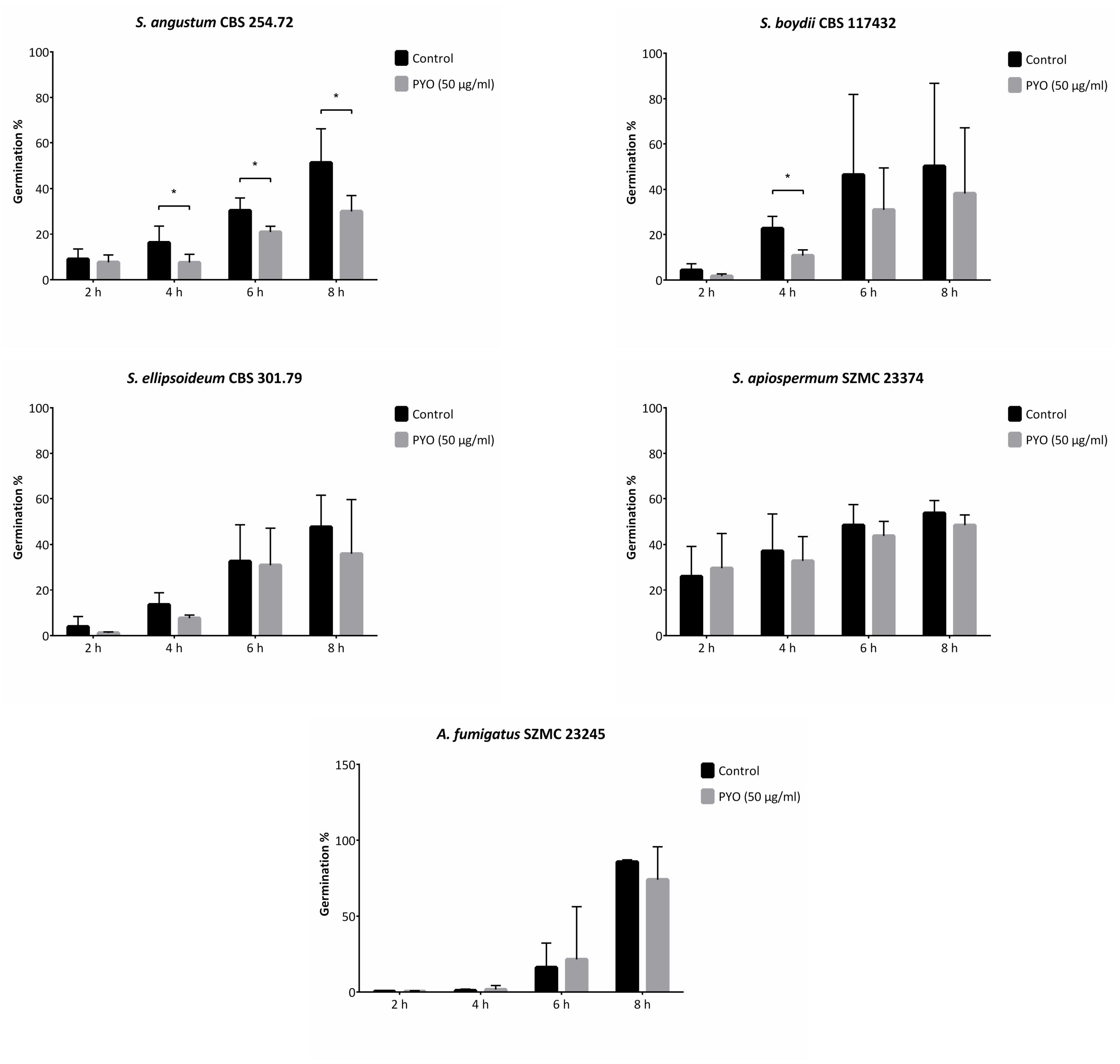

Figure 4S Germination ability of conidia collected from *Scedosporium* and *A. fumigatus* isolates in the presence of 50 µg/ml pyocyanin. Results are the mean of three independent experiments. Error bars indicate standard deviations. P values were calculated using unpaired t test, where $p<0.05$ was considered to be significant (indicated with asterisks).

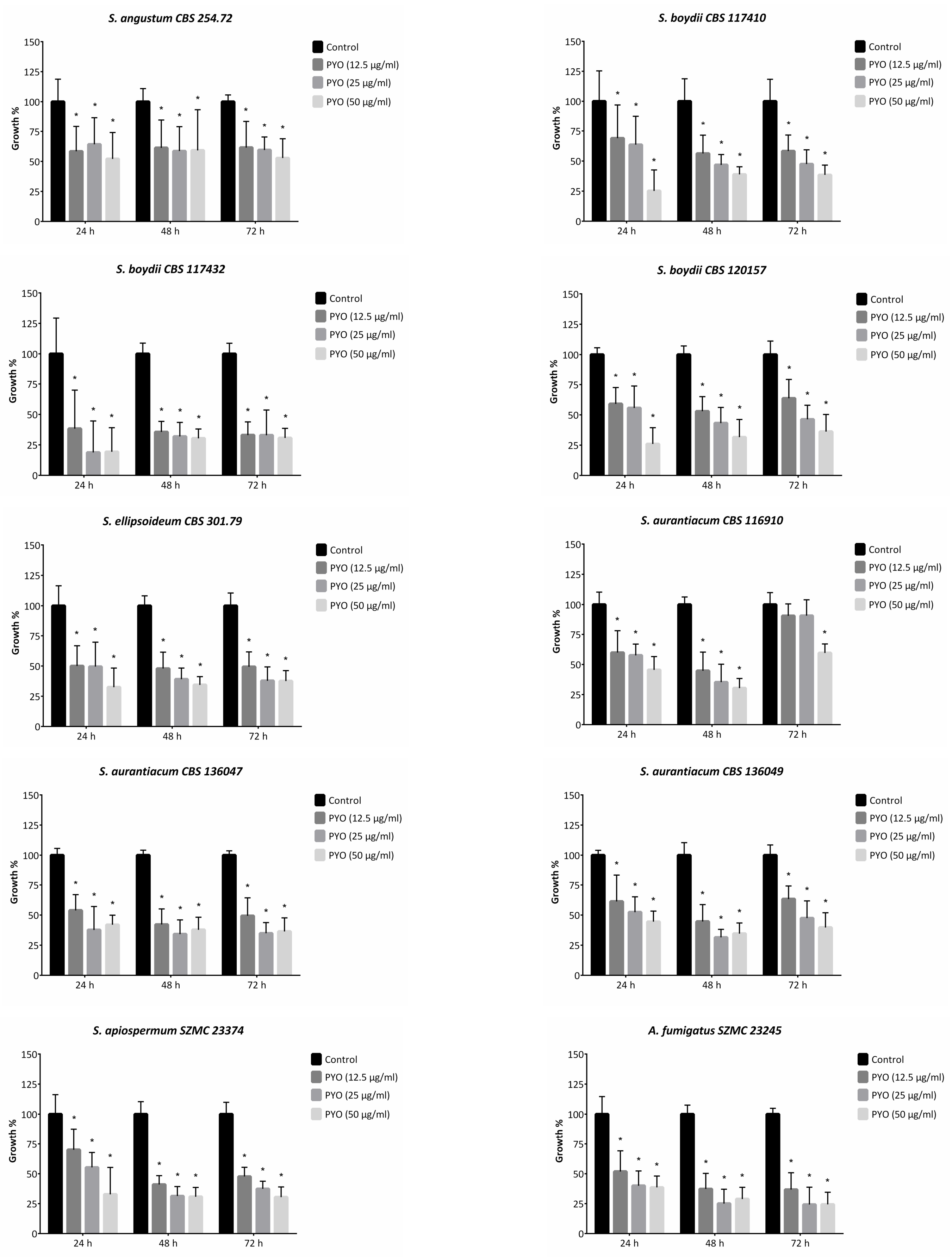

Figure 5S Growth rate of *Scedosporium* and *A. fumigatus* isolates in the presence of 50 µg/ml pyocyanin. Results are the mean of three independent experiments with three individual replicates. Error bars indicate standard deviations. P values were calculated using unpaired t test, where $p<0.05$ was considered to be significant (indicated with asterisks).

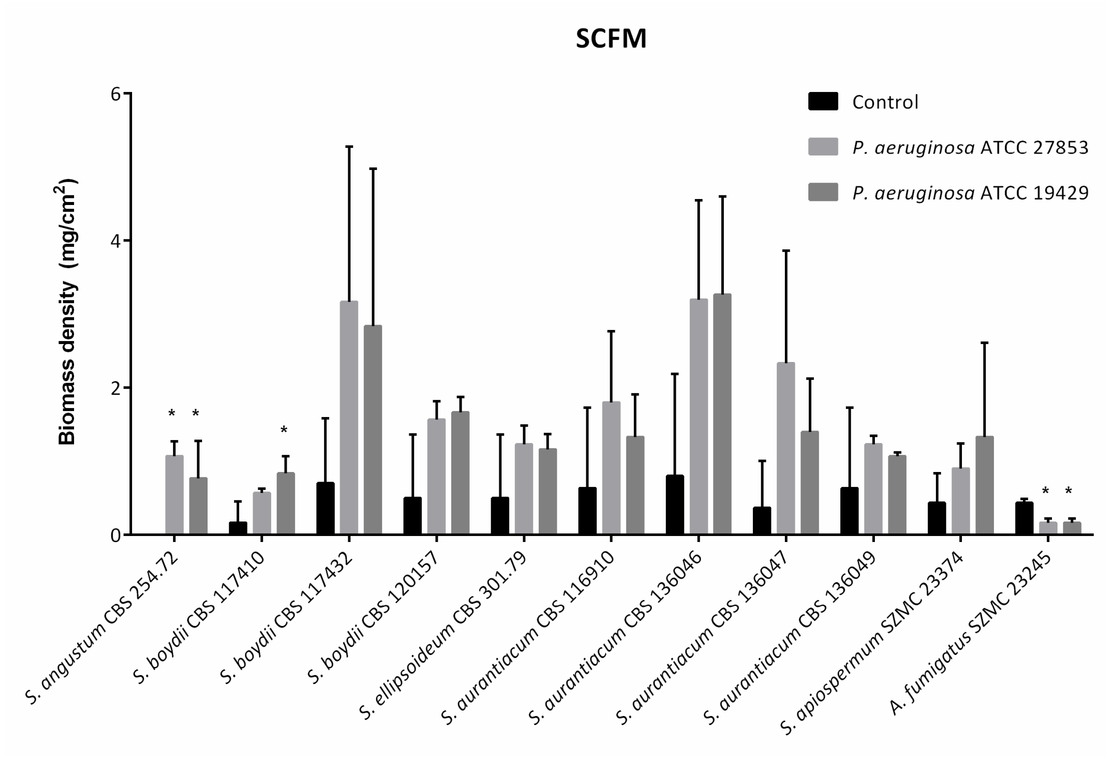

Figure 6S The biomass densities of *Scedosporium* and *Aspergillus fumigatus* isolates grown on synthetic cystic fibrosis medium (SCFM) in the plate-in-plate tests in absence (control) and in the presence of *Pseudomonas aeruginosa* strains ATCC 27853 and 19429. Results are the mean of three independent experiments. Error bars indicate standard deviations. P values were calculated using unpaired t test, where $p<0.05$ was considered to be significant (indicated with asterisks).

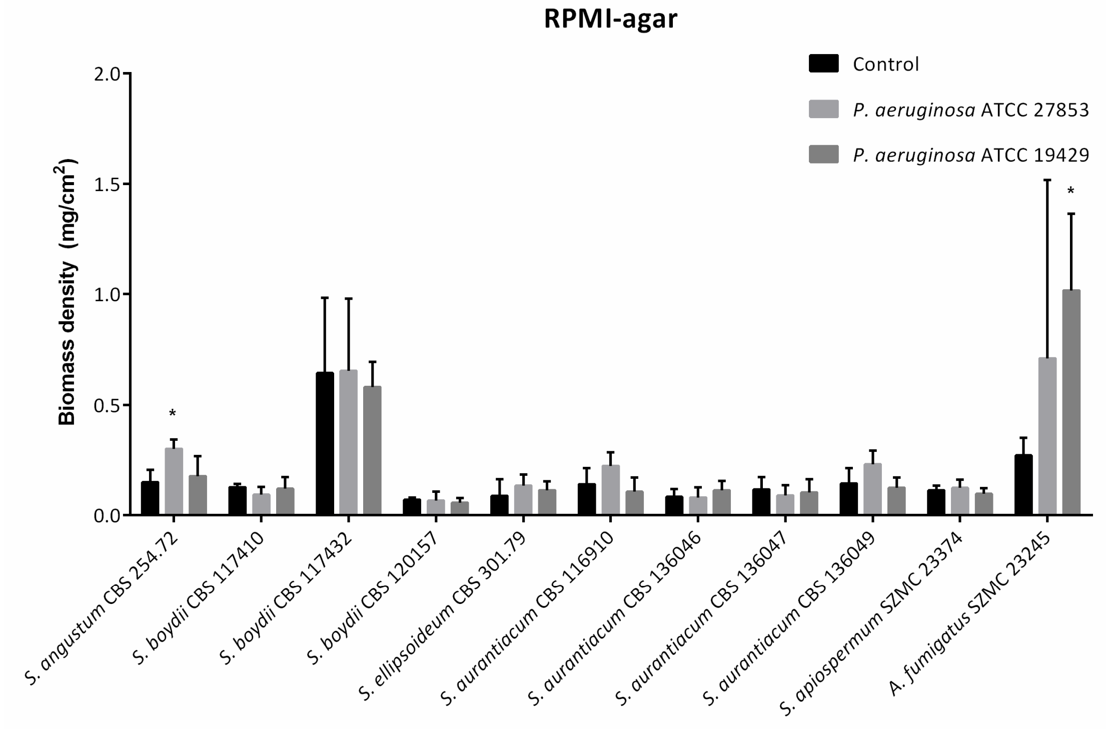

Figure 7S The biomass densities of *Scedosporium* and *Aspergillus fumigatus* isolates grown on RPMI-agar in the plate-in-plate tests in absence (control) and in the presence of *Pseudomonas aeruginosa* strains ATCC 27853 and 19429. Results are the mean of three independent experiments. Error bars indicate standard deviations. P values were calculated using unpaired t test, where $p<0.05$ was considered to be significant (indicated with asterisks).

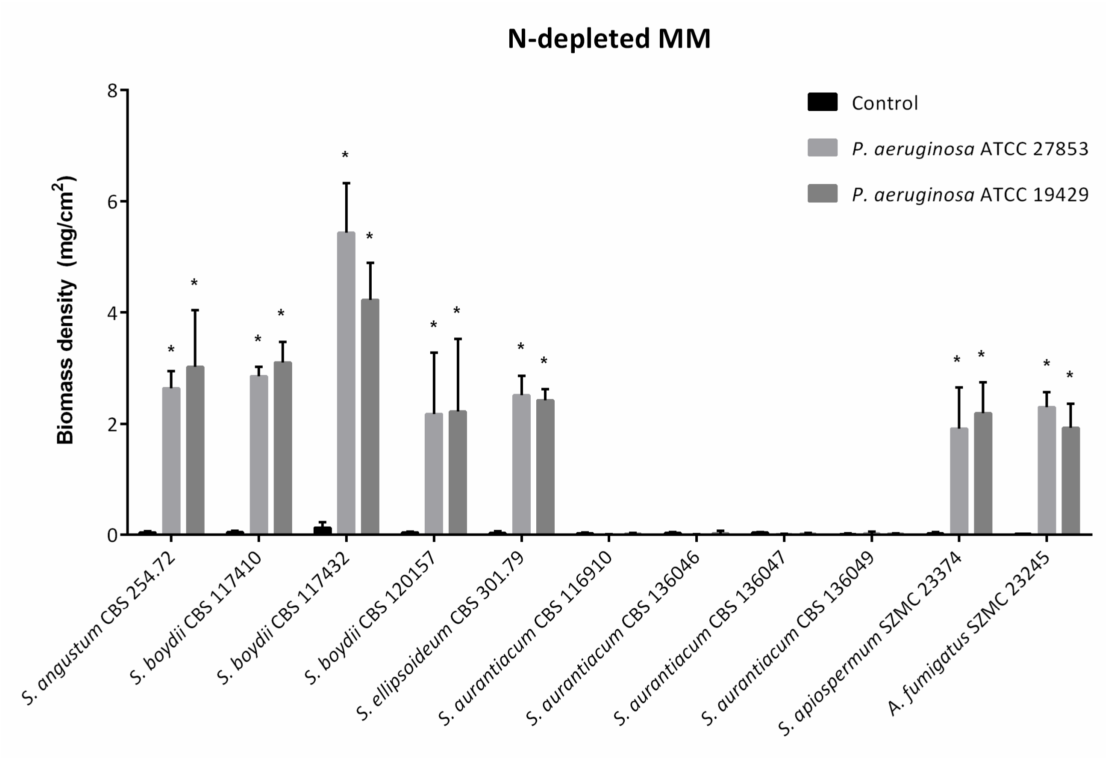

Figure 8S The biomass densities of *Scedosporium* and *Aspergillus fumigatus* isolates grown on nitrogen-depleted minimal medium (N-depleted MM) in the plate-in-plate tests in absence (control) and in the presence of *Pseudomonas aeruginosa* strains ATCC 27853 and 19429. Results are the mean of three independent experiments. Error bars indicate standard deviations. P values were calculated using unpaired t test, where $p<0.05$ was considered to be significant (indicated with asterisks).

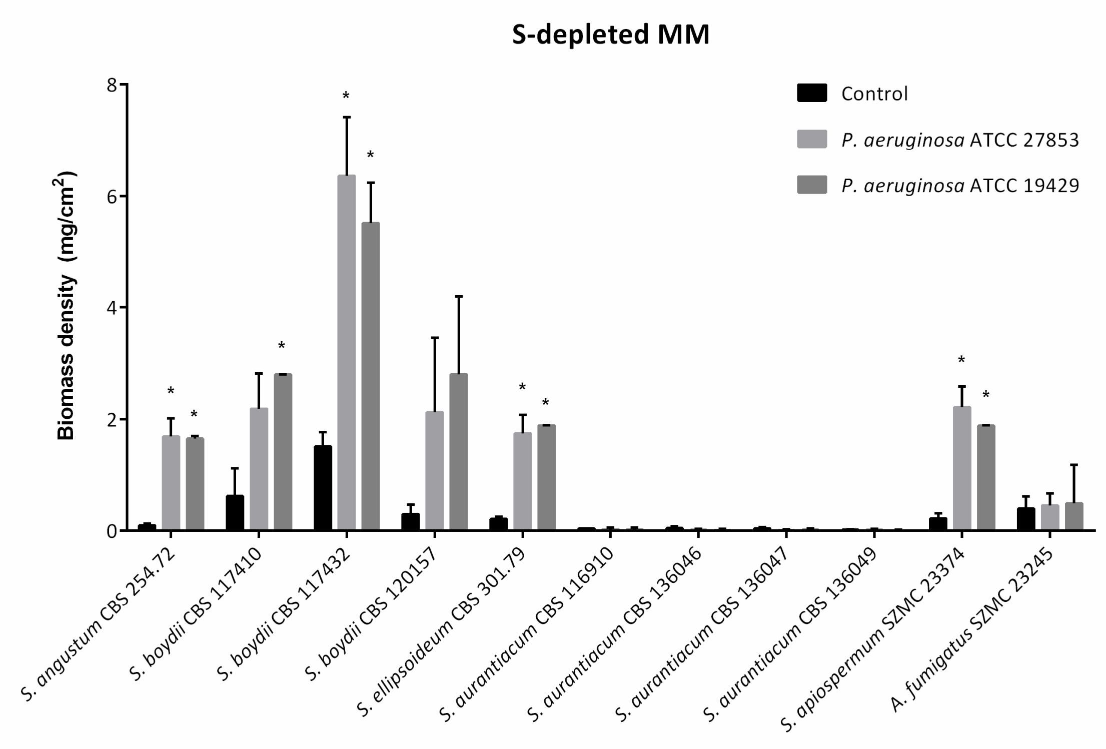

Figure 9S The biomass densities of *Scedosporium* and *Aspergillus fumigatus* isolates grown on sulfur-depleted minimal medium (S-depleted MM) in the plate-in-plate tests in absence (control) and in the presence of *Pseudomonas aeruginosa* strains ATCC 27853 and 19429. Results are the mean of three independent experiments. Error bars indicate standard deviations. P values were calculated using unpaired t test, where $p<0.05$ was considered to be significant (indicated with asterisks).

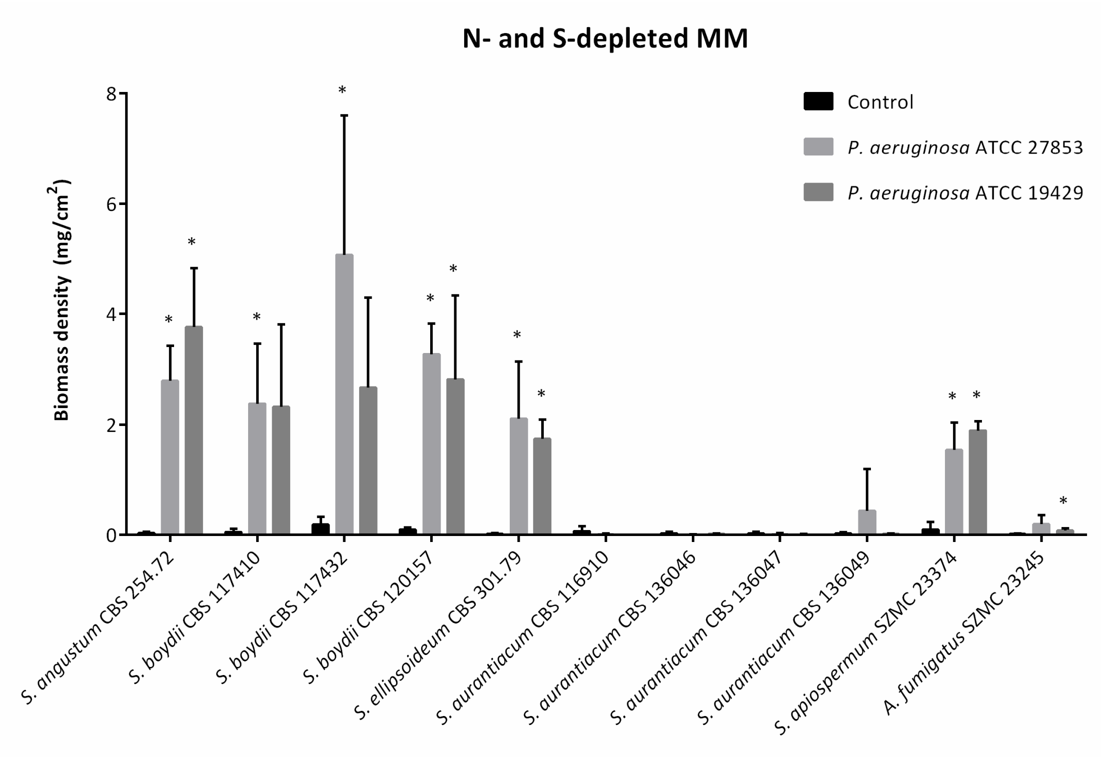

Figure 10S The biomass densities of *Scedosporium* and *Aspergillus fumigatus* isolates grown on nitrogen- and sulfur-depleted minimal medium (N- and S-depleted MM) in the plate-in-plate tests in absence (control) and in the presence of *Pseudomonas aeruginosa* strains ATCC 27853 and 19429. Results are the mean of three independent experiments. Error bars indicate standard deviations. P values were calculated using unpaired t test, where p<0.05 was considered to be significant (indicated with asterisks).

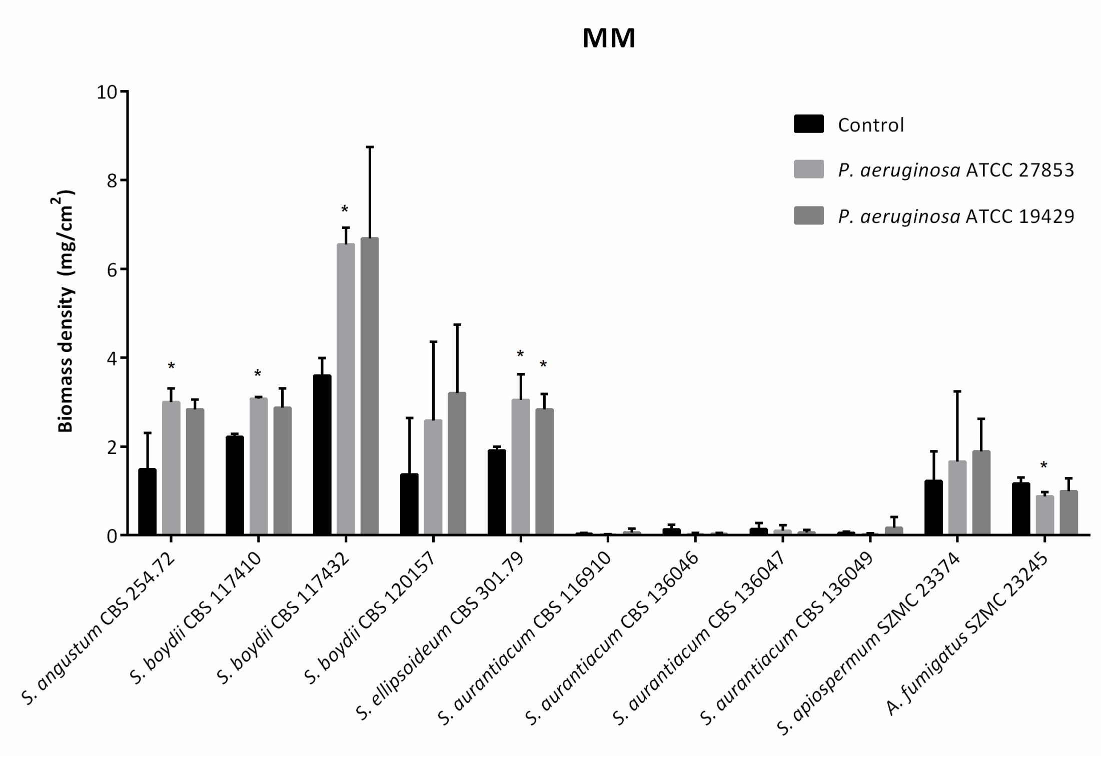

Figure 11S The biomass densities of *Scedosporium* and *Aspergillus fumigatus* isolates grown on minimal medium (MM) in the plate-in-plate tests in absence (control) and in the presence of *Pseudomonas aeruginosa* strains ATCC 27853 and 19429. Results are the mean of three independent experiments. Error bars indicate standard deviations. P values were calculated using unpaired t test, where p<0.05 was considered to be significant (indicated with asterisks).

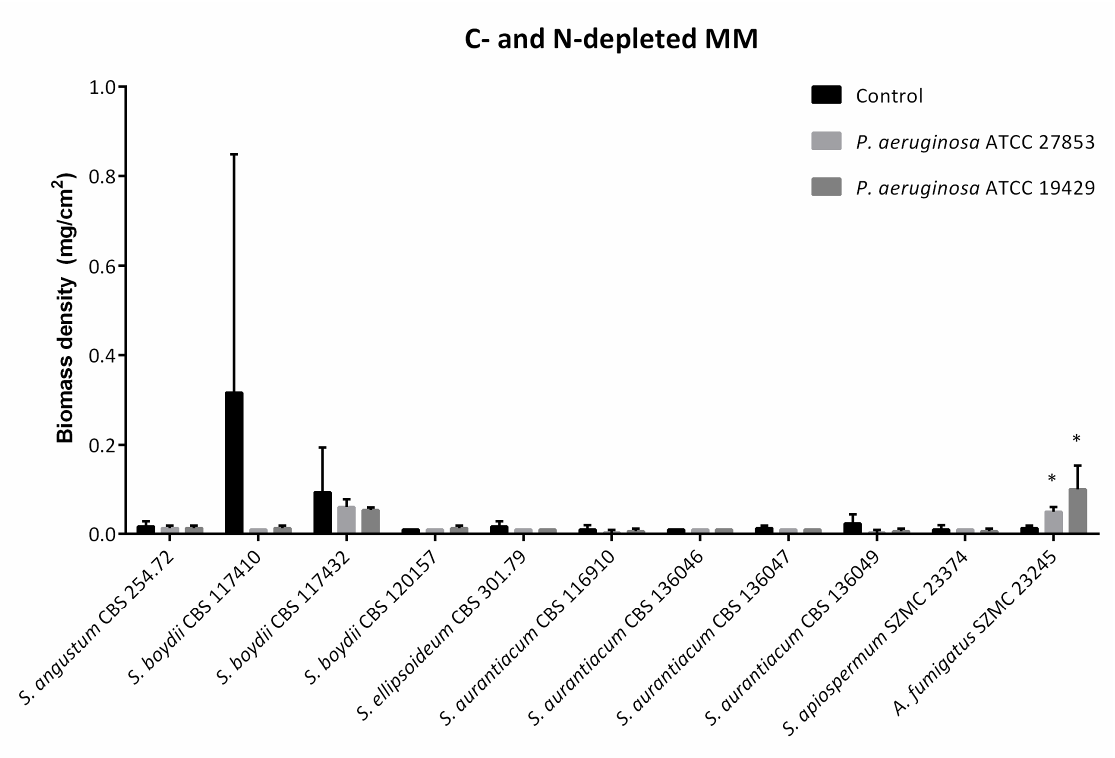

Figure 12S The biomass densities of *Scedosporium* and *Aspergillus fumigatus* isolates grown on carbon- and nitrogen-depleted minimal medium (C- and N-depleted MM) in the plate-in-plate tests in absence (control) and in the presence of *Pseudomonas aeruginosa* strains ATCC 27853 and 19429. Results are the mean of three independent experiments. Error bars indicate standard deviations. P values were calculated using unpaired t test, where $p<0.05$ was considered to be significant (indicated with asterisks).

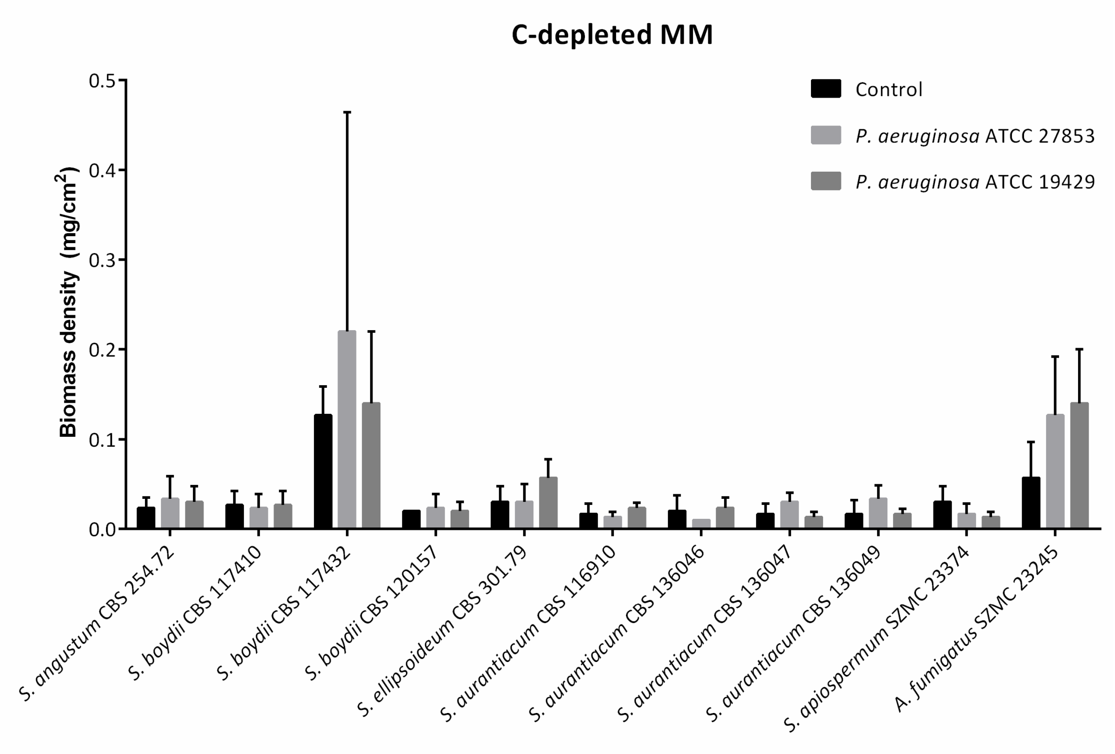

Figure 13S The biomass densities of *Scedosporium* and *Aspergillus fumigatus* isolates grown on carbon-depleted minimal medium (C-depleted MM) in the plate-in-plate tests in absence (control) and in the presence of *Pseudomonas aeruginosa* strains ATCC 27853 and 19429. Results are the mean of three independent experiments. Error bars indicate standard deviations. P values were calculated using unpaired t test, where $p<0.05$ was considered to be significant (indicated with asterisks).

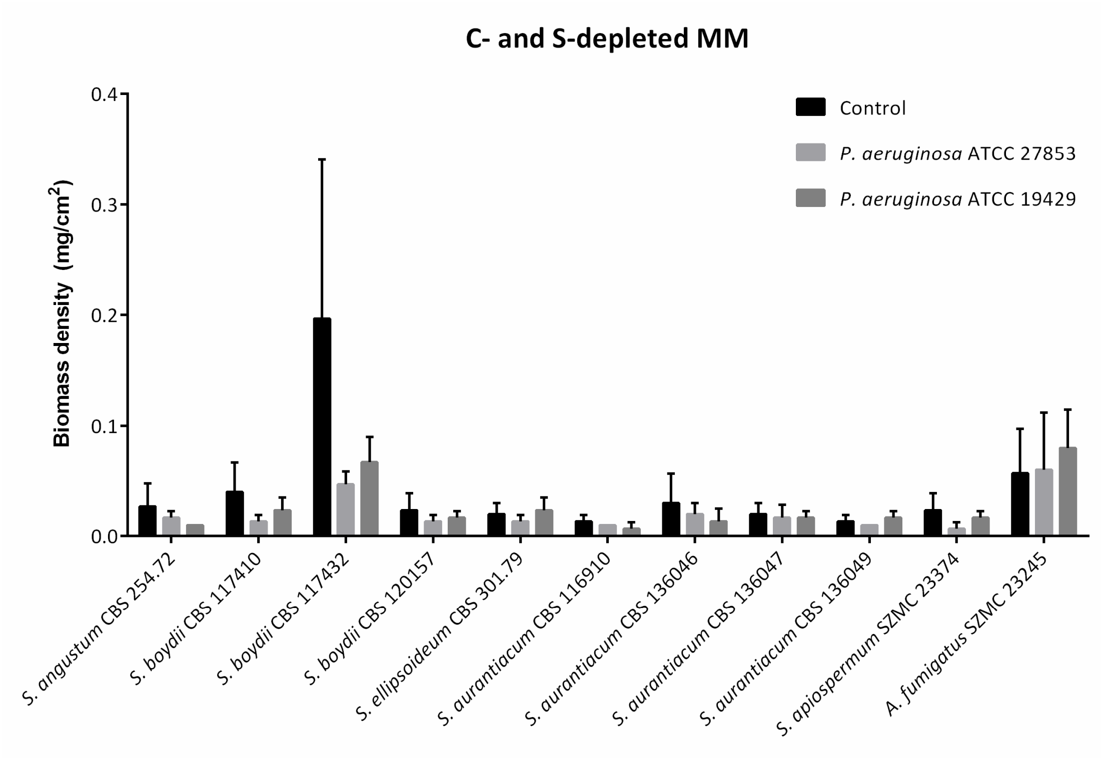

Figure 14S The biomass densities of *Scedosporium* and *Aspergillus fumigatus* isolates grown on carbon- and sulfur-depleted minimal medium (C- and S-depleted MM) in the plate-in-plate tests in absence (control) and in the presence of *Pseudomonas aeruginosa* strains ATCC 27853 and 19429. Results are the mean of three independent experiments. Error bars indicate standard deviations. P values were calculated using unpaired t test, where $p<0.05$ was considered to be significant (indicated with asterisks).

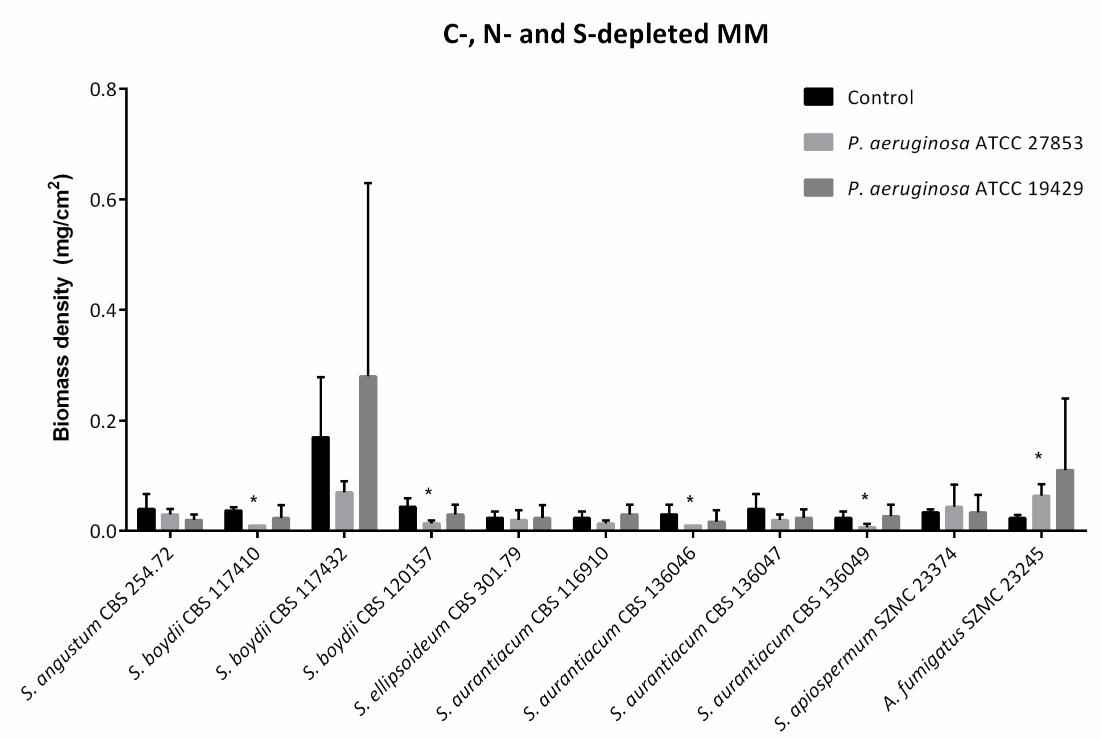

Figure 15S The biomass densities of *Scedosporium* and *Aspergillus fumigatus* isolates grown on carbon-, nitrogen and sulfur-depleted minimal medium (C-, N- and S-depleted MM) in the plate-in-plate tests in absence (control) and in the presence of *Pseudomonas aeruginosa* strains ATCC 27853 and 19429. Results are the mean of three independent experiments. Error bars indicate standard deviations. P values

were calculated using unpaired t test, where $p<0.05$ was considered to be significant (indicated with asterisks).

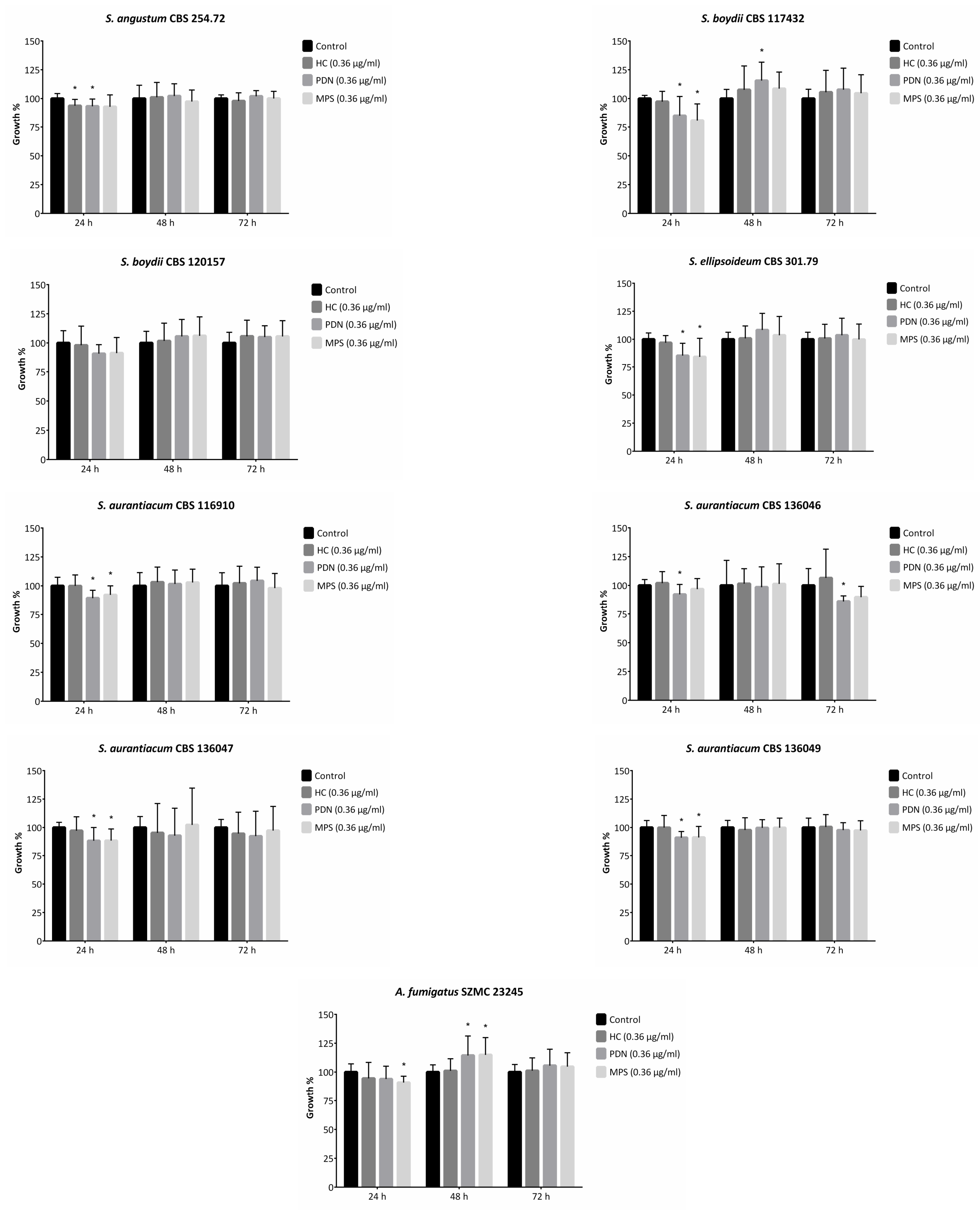

Figure 16S Growth rate of *Scedosporium* and *A. fumigatus* isolates in the presence of 10^{-6} M (approx. 0.36 µg/ml) corticosteroid compounds (*i.e.*, hydrocortisone, HC; prednisone, PDN; methylprednisolone, MPS). Results are the mean of three independent experiments with three individual replicates. Error bars indicate standard deviations. P values were calculated using unpaired t test, where $p<0.05$ was considered to be significant (indicated with asterisks).

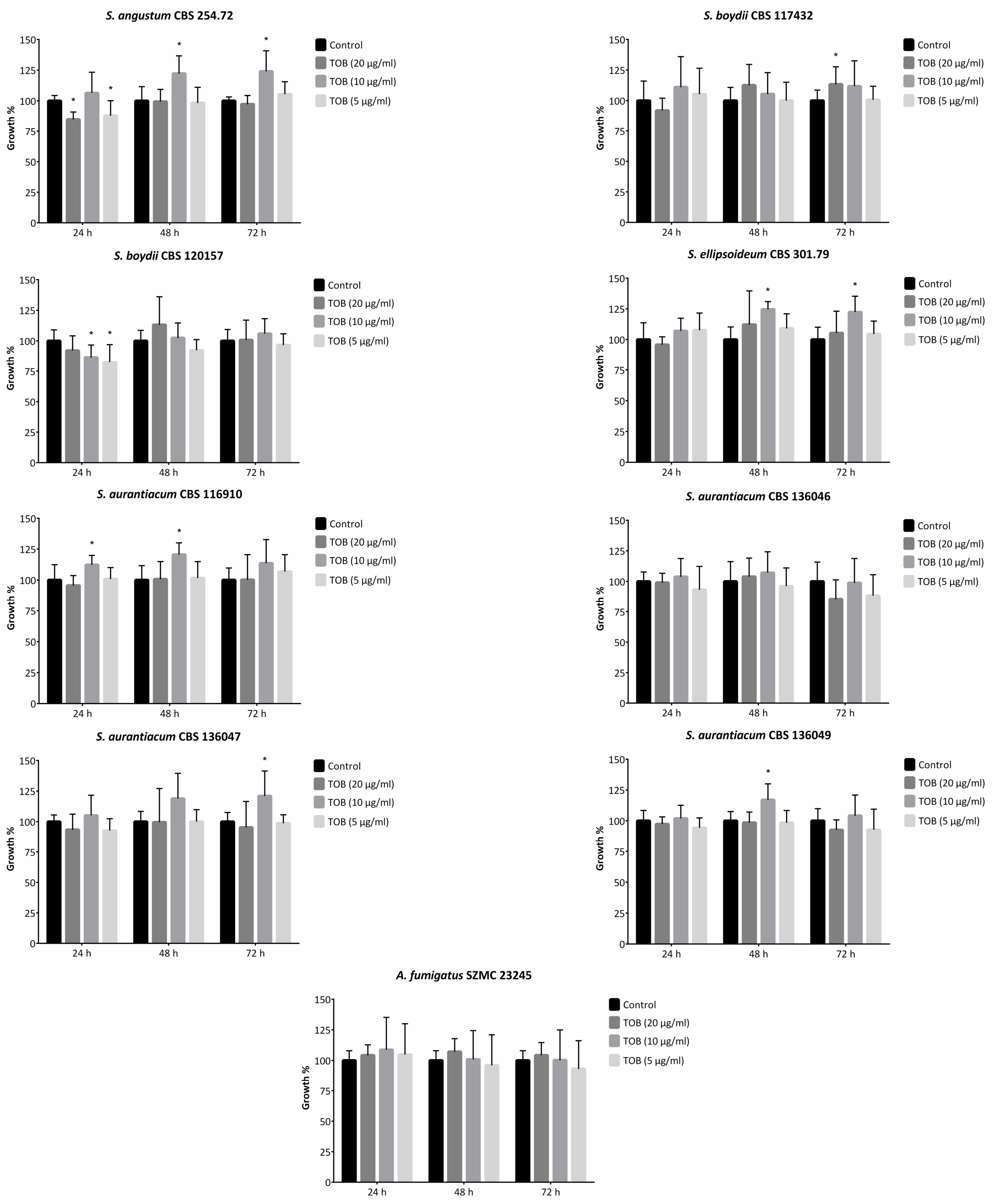

Figure 17S Growth rate of *Scedosporium* and *A. fumigatus* isolates in the presence of tobramycin (TOB). Results are the mean of three independent experiments with three individual replicates. Error bars indicate standard deviations. P values were calculated using unpaired t test, where $p<0.05$ was considered to be significant (indicated with asterisks).

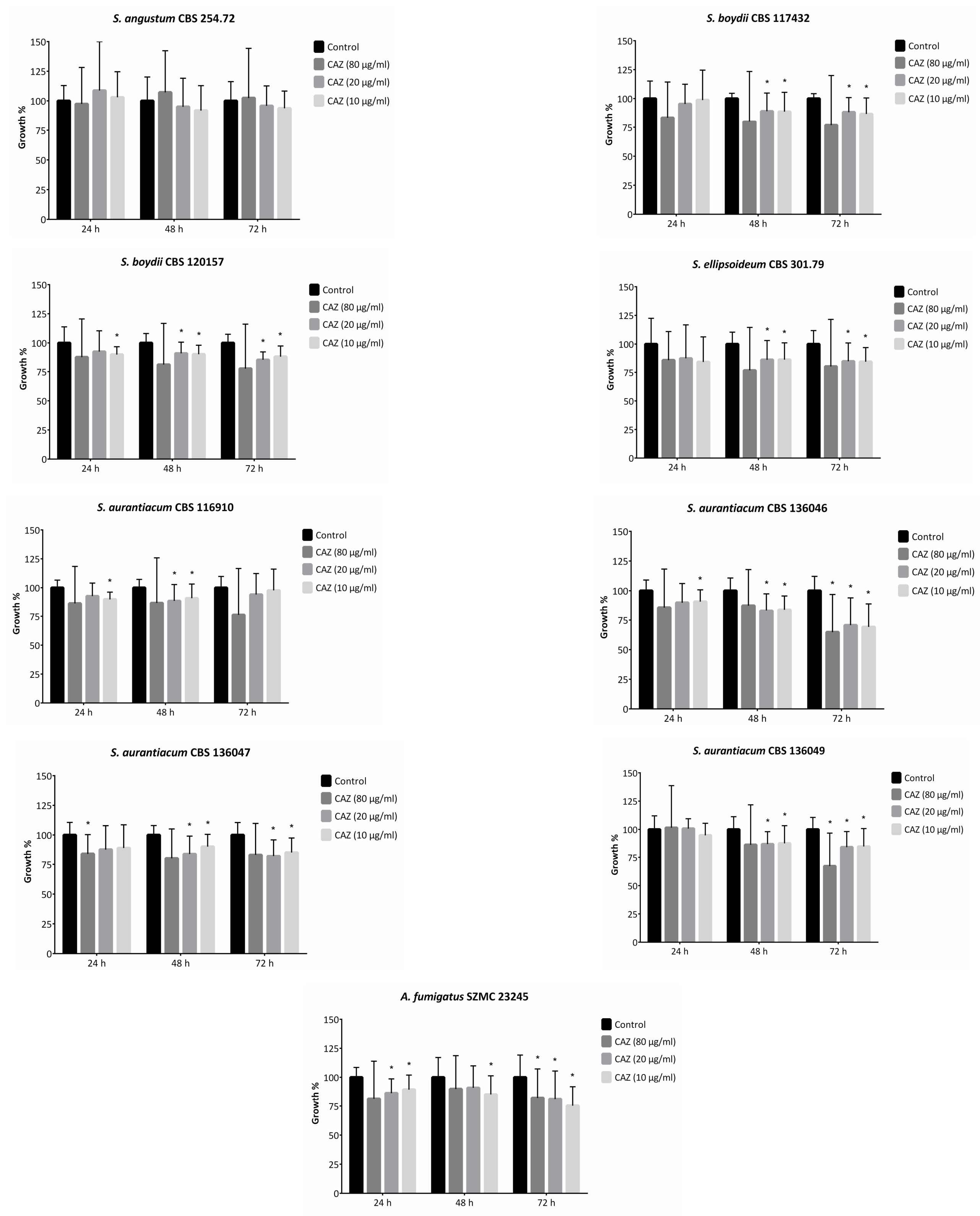

Figure 18S Growth rate of *Scedosporium* and *A. fumigatus* isolates in the presence of ceftazidime (CAZ). Results are the mean of three independent experiments with three individual replicates. Error bars indicate standard deviations. P values were calculated using unpaired t test, where $p<0.05$ was considered to be significant (indicated with asterisks).

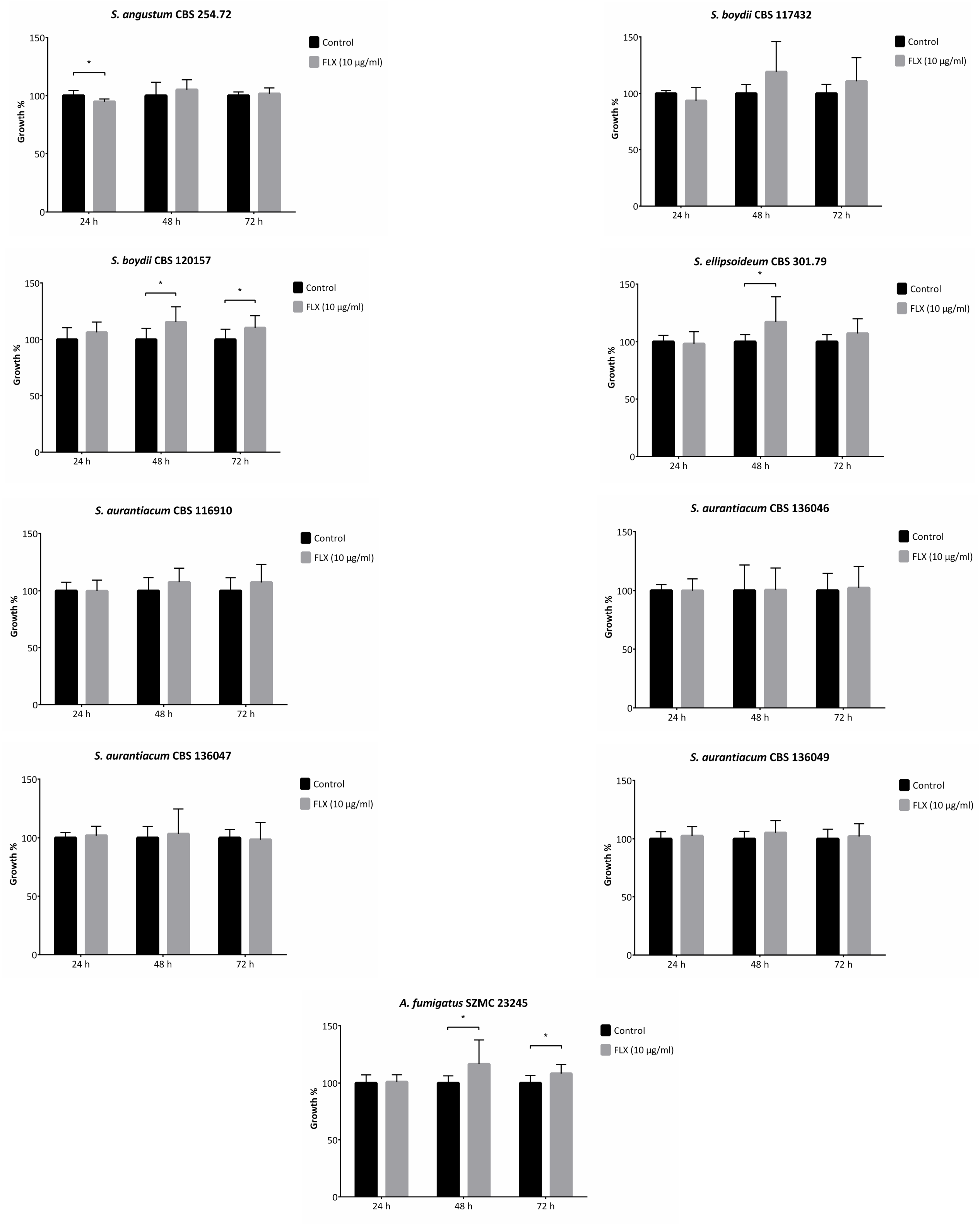

Figure 19S Growth rate of *Scedosporium* and *A. fumigatus* isolates in the presence of 10 µg/ml flucloxacillin (FLX). Results are the mean of three independent experiments with three individual replicates. Error bars indicate standard deviations. P values were calculated using unpaired t test, where $p<0.05$ was considered to be significant (indicated with asterisks).
